# Ultra‐Sensitive and Linear Flexible Pressure Sensors with Tri‐Scale Graded Microstructures for Advanced Health Monitoring and Robotic Perception

**DOI:** 10.1002/advs.202516810

**Published:** 2025-10-20

**Authors:** Rui Chen, Chi Fai Cheung, Qixian Zhang, Tao Luo, Rui Gao, Wei Zhou, Chunjin Wang

**Affiliations:** ^1^ State Key Laboratory of Ultra‐Precision Machining Technology Department of Industrial and Systems Engineering The Hong Kong Polytechnic University Hong Kong Kowloon China 999077; ^2^ Pen‐Tung Sah Institute of Micro‐Nano Science and Technology Xiamen University Xiamen 361102 China

**Keywords:** flexible piezoresistive sensor, graded microstructures, high sensitivity, human‐machine interaction, laser processing

## Abstract

Flexible piezoresistive sensors, which combine high sensitivity and a wide linear detection range, are ideal choices for human health monitoring and robotic perception. However, sensors often exhibit a trade‐off between sensitivity and linearity, with challenges caused by the incompressibility of soft materials and the stiffening of microstructures. In this study, a flexible pressure sensor with a 3D ordered tri‐scale graded microstructure, fabricated by laser processing, is proposed. The sensor achieves an ultra‐high sensitivity of 138.6 kPa^−1^ and a linear range up to 400 kPa (*R* ^2^ = 0.99). The compensation behavior derived from the tri‐scale graded microstructure's compression deformation counteracts contact hardening and delays sensitivity saturation. Furthermore, the sensor demonstrates a minimum detectable limit as low as 3 Pa, with response and recovery times of 34/39 ms, showing excellent stability after over 24 000 repeated loading cycles. Physiological monitoring confirms that the sensor can accurately capture a wide range of pressure‐variations, including those from the carotid artery, jugular vein, respiration, throat vibrations, and foot pressure. Additionally, the sensor can be used for remote operation of robotic hands. This work provides a strategy for manufacturing flexible pressure sensors with a combination of high sensitivity, high linearity, and a wide pressure response range.

## Graphical abstract

A flexible piezoresistive sensor with a 3D tri‐scale graded microstructure is proposed, overcoming the trade‐off between sensitivity and linearity. The device achieves ultra‐high sensitivity (138.6 kPa^−1^), a broad linear range (400 kPa, *R* ^2^ = 0.99), and exceptional stability (24 000 repeated loading cycles), enabling precise detection of physiological signals and robotic interactions. This study offers a new strategy for advanced health monitoring and intelligent robotics.

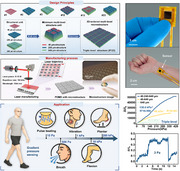

## 1 Introduction

Flexible piezoresistive sensors, which convert mechanical stimuli into electrical signals, are crucial for wearable health monitoring and human‐machine interaction applications.^[^ ^1^, ^2^, ^3^ ^]^ Compared to capacitive and piezoelectric sensors, piezoresistive sensors are widely studied due to their stable signal output, simple structure, and low cost. While progress has been made in sensitivity, detection limit, and stability of flexible piezoresistive pressure sensors, a key challenge in practical applications is balancing high sensitivity (>10 kPa^−1^) with a wide linear detection range (>100 kPa, *R* ^2^ > 0.99).^[^ ^4^, ^5^, ^6^, ^7^, ^8^, ^9^ ^]^ High sensitivity enables detection of subtle pressure changes, while a broad detection range allows the sensor to handle both low and high‐pressure signals, eliminating the need for multiple sensors.^[^ ^10^, ^11^ ^]^ Furthermore, the linear signal characteristics also simplify data analysis without requiring complex processing. For physiological signal monitoring, the sensor must maintain high sensitivity under both low pressure (<1 kPa) and high pressure (>200 kPa), while covering common physiological pressures (e.g., pulse pressure 1–10 kPa, foot pressure 100–300 kPa).^[^ ^12^, ^13^ ^]^ Thus, maintaining high sensitivity while extending the linear detection range remains a major research focus.

Microstructure design has emerged as a key strategy to address sensitivity and linear response challenges for flexible sensors.^[^ ^14^, ^15^, ^16^ ^]^ Studies have shown that the microstructure of the pressure‐sensing layer can improve material compressibility and pressure dependence on the contact area. Some researchers have proposed single‐level microstructures, such as micro‐pyramid arrays,^[^ ^17^, ^18^, ^19^ ^]^ domed,^[^ ^20^, ^21^ ^]^ cylindrical,^[^ ^22^, ^23^ ^]^ porous structures,^[^ ^24^, ^25^ ^]^ and natural prototypes (e.g., plant leaves),^[^ ^26^, ^27^ ^]^ to enhance sensitivity. However, these designs are usually effective only within a low‐to‐medium pressure range (50–100 kPa), with sensitivity decreasing at higher pressures (>200 kPa). Despite significant research on microstructure‐based pressure sensors, balancing sensitivity and linear range remains challenging.^[^ ^28^ ^]^ The factors limiting performance can be summarized as: (1) rapid contact saturation, limiting the linear sensing range; (2) high‐pressure densification, reducing sensitivity and responsiveness; and (3) instability of microstructures causing hysteresis, signal drift, and mechanical fatigue. Achieving high sensitivity and linear response depends on the interaction between geometric deformation and signal transduction. Therefore, microstructure engineering is essential for sensor linearization.

To overcome these challenges, researchers have proposed multilevel microstructure designs, enabling differentiated responses across various pressure ranges.^[^ ^29^, ^30^ ^]^ Multilevel microstructures, spanning multiple scales, form a synergistic effect. Geng et al.^[^ ^31^ ^]^ used laser processing to create spherical microstructure arrays of different sizes, delaying sensitivity saturation, with three‐level microstructures offering twice the sensitivity of single‐level structures. Xu et al.^[^ ^32^ ^]^ proposed a forward design strategy based on hyperelastic and Hertzian contact models,^[^ ^33^, ^34^ ^]^ achieving customizable sensitivity and linearity via 3D printing of graded hemispherical microstructure arrays. Although multilevel microstructure designs improve sensor performance, challenges remain in optimizing linearity and simplifying fabrication. Thus, designing easy‐to‐fabricate graded microstructures and developing fabrication methods is critical for sensor linearization.^[^ ^35^ ^]^ By establishing a cooperative regulation mechanism between physical deformation and interface contact, more uniform pressure transfer can be achieved from local deformation to the overall sensor interface. This approach mitigates the compression hardening effect of microstructures, delaying signal saturation at high pressures, while maintaining sensitivity in the high‐pressure range, ultimately achieving linear sensor responses.

Here, we present a flexible piezoresistive pressure sensor based on a 3D ordered tri‐scale graded microstructure array (denoted as 3D‐OTGM), in which a polyethylene oxide/carbon black (CB@PEO) composite is employed as the sensing material. The sensor is fabricated by laser processing to form the tri‐scale graded architecture. Under high pressure, when the small‐scale microstructures reach deformation saturation, the larger‐scale structures continue to deform and create new contact areas. This graded mechanism enables high sensitivity (138.6 kPa^−1^) and excellent linearity (*R* ^2^ > 0.99) within a wide pressure range of up to 400 kPa. The unique structural design enhances the compressibility of the sensing material while mitigating the hardening effect often observed in microstructured systems. The device also delivers a rapid response and recovery time of less than 39 ms, an ultralow detection limit of 3 Pa, and outstanding durability over 24 000 loading cycles at 70 kPa. In addition to these performance metrics, the 3D‐OTGM sensor demonstrates versatility in practical applications. It reliably records various physiological signals such as respiration, carotid, venous, and wrist pulses, finger bending, and plantar pressure. Furthermore, it functions as a human‐machine interface for robotic hand gesture control. More broadly, the laser‐fabricated graded microstructure strategy provides a generalizable design principle that can be adapted to different material systems and sensing mechanisms.

## 2 Results and Discussion

### 2.1 Design Principles of 3D Ordered Tri‐Scale Graded Microstructure

The compressibility of the sensor interface microstructure directly determines its sensing characteristics. It has been demonstrated that a properly designed microstructure can effectively regulate the sensor's sensitivity and pressure response range. According to classical contact mechanics, including Archard theory.^[^ ^33^ ^]^ and Hertzian theory,^[^ ^34^ ^]^ a linear relationship exists between the actual contact area (*A*_real_) and the applied pressure (*P*), i.e., *A*_real_∝*P*. Since the electrical output signal (*S*) is proportional to the actual contact area, *S*∝*A*_real_, combining these two relationships results in a linear pressure signal response: *S*∝*A*_real_∝*P*, meaning that sensitivity (d*S*/d*P*) is constant. Therefore, to achieve a pressure sensor with high sensitivity and a wide range, the 3D ordered tri‐scale graded microstructure should satisfy the following requirements: At low pressure, the small structures undergo deformation saturation first; as pressure increases, although the small structures are saturated, the larger structures continue to deform, generating new contact areas, thus increasing the contact area. This continuous structural deformation maintains a high interface compressibility and enables the contact area to increase in a stable and nearly linear manner across a wide pressure range, thereby enhancing the sensitivity, linearity, and range of the sensor.

**Figure 1:**
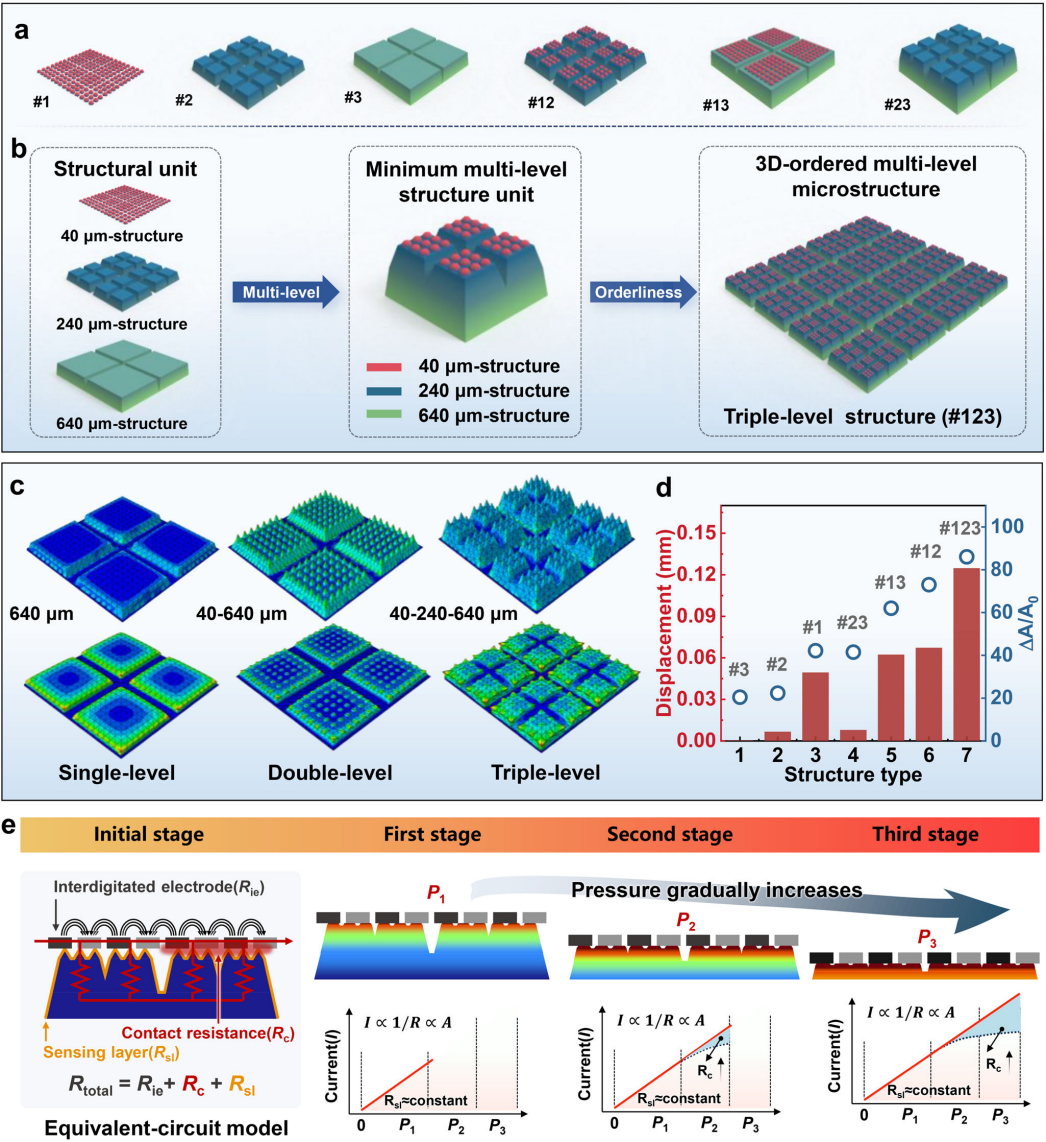
Design principles and sensing mechanism of the 3D ordered tri‐scale graded microstructure. a) Schematic of single‐level and double‐level microstructure compositions. b) Schematic of the minimal structural unit and construction of the 3D ordered tri‐scale graded microstructure. c) Finite element simulation results showing the compression of the microstructure under 4 N pressure. d) Compression displacement and contact area change rate for seven types of microstructures. e) Contact resistance model and schematic showing the current change with pressure increase at different pressure stages.

To study the effects of different microstructure grades on performance, we designed basic single‐level microstructure units, as shown in **Figure** **1**a,b. The single‐level microstructures have dimensions of 40, 240, and 640 µm, defined as #1, #2, and #3, respectively. By combining two basic units, we constructed a double‐level microstructure array, including #12 (40–240 µm), #13 (40–640 µm), and #23 (240–640 µm). Further, by combining all three single‐level microstructure units, we constructed a triple‐level microstructure, defined as #123 (40‐240‐640 µm). By arranging these triple‐level microstructures in an ordered fashion, we obtained a 3D ordered tri‐scale graded microstructure array, as shown in Figure 1b. The selection of 40 µm as the smallest fundamental unit was determined by the resolution limit of our laser fabrication process, which is ≈40 µm. Structures smaller than this threshold cannot be produced with sufficient clarity and controllability. To construct a 3 × 3 array of primary units within the secondary structure, a width of ≈200 µm is required to account for the spacing between adjacent microstructures. Therefore, a 240 µm secondary unit was designed to ensure sufficient tolerance. Similarly, to form a 2 × 2 array of secondary units within the tertiary structure, a 640 µm tertiary unit was selected. If a smaller primary unit size were chosen, it would exceed the fabrication resolution, leading to incomplete or unstable structures. Conversely, excessively large units would disrupt the hierarchical nesting relationship, thereby reducing sensitivity and spatial efficiency. Thus, the dimensions of 40/240/640 µm represent a rational compromise between fabrication feasibility, hierarchical structural logic, and sensing performance.

Finite element analysis (FEA) was employed to investigate the mechanical properties and contact interface configurations of seven microstructure arrays formed by single‐level microstructure units. The pressure sensor consists of a PDMS substrate and a thin CB@PEO film that serves as the pressure‐sensing layer. Because the thickness of the CB@PEO film is negligible compared with the size of the microstructures, its influence on the overall mechanical behavior was disregarded. Accordingly, the finite element model was simplified to PDMS, with a Young's modulus of 1.23 MPa and a Poisson's ratio of 0.48. As the simulated strain range was relatively small, a linear elastic model was adopted rather than a more complex hyperelastic model.

For the boundary conditions, the bottom surface of the microstructure was fully fixed, while a rigid plane was placed at the top and defined as an analytical rigid body to apply the load. Surface‐to‐surface contact was established between the PDMS and the rigid plane, and Coulomb friction with a coefficient of 0.2 was applied. A load of 4 N was imposed on the rigid plane to simulate the deformation of the microstructures. From the simulation, the von Mises stress and displacement distributions were obtained, together with the displacement of the rigid body reference point. The corresponding change in contact area (Δ*A*_c_) was also extracted to analyze the pressure response of the microstructure interface, as illustrated in Figure 1c,d.

As the number of structural grades increases, the relative structural strength decreases, and the displacement deformation increases under the same pressure. Therefore, the triple‐level microstructure exhibits the largest deformation, followed by the double‐level structure, with the single‐level structure exhibiting the smallest deformation. The analysis shows that the change in contact area is the decisive factor in the variation of sensor resistance during structural deformation.^[^ ^36^ ^]^ The interface compressibility of the single‐level microstructure is poor, resulting in a small change in contact area (Δ*A*/*A*_0_), where *A*_0_ is the initial contact area. The contact area change rate for #1 is significantly higher than for #2 and #3 because #1 has a smaller initial contact area. The compressibility of the double‐level microstructure array is significantly improved, and the corresponding Δ*A*/*A*_0_ also increases. The compressibility of the triple‐level microstructure is the highest, and the contact area change rate is also the largest (Figure 1d). Therefore, designing a 3D ordered tri‐scale graded microstructure can effectively regulate the sensitivity and linear range of the sensor.

To further understand the relationship between resistance response and contact area changes, we developed a simplified circuit model, as shown in Figure 1e. The total resistance (*R*_total_) of the piezoresistive sensor consists of three parts: the interdigitated electrode resistance (*R*_ie_), the conductive film layer resistance of the microstructure (*R*_sl_), and the contact resistance (*R*_c_):

(1) $$R_{\mathrm{total}}=R_{\mathrm{ie}}+R_{\mathrm{sl}}+R_{c}$$

Since the interdigitated electrode has good conductivity, and its resistance was measured to be less than 3 Ω (Figure S1, Supporting Information), with almost no change observed under bending conditions. By contrast, the resistance of the CB@PEO conductive layer is in the 50‐60 megaohm range (Figure S2, Supporting Information), which is approximately five orders of magnitude higher. Therefore, *R*_ie_ can be neglected. Therefore, this equation can be simplified to:

(2) $$R_{total}=R_{sl}+R_{c}$$

Here, *R*_sl_ remains nearly constant, and the contact resistance *R*_c_ is inversely proportional to the contact area *A* and current density *J*.^[^ ^30^ ^]^ In the initial state, only a limited number of microcones at the interface between the microstructure and electrode are in contact, resulting in a minimal initial contact area (*A*_0_), leading to a high *R*_c_ and low initial current (*I*_0_). When a low pressure is applied, more microstructures deform, gradually increasing the contact area. In the medium pressure range (*P*_2_), the double‐level microstructure continues to compress, further increasing the contact area and compensating for the decreasing electrical signal. At high pressure (*P*_3_), the triple‐level microstructure is compressed, continuously generating new contact points and further delaying the saturation of the electrical signal curve. As a result, the current response remains approximately linear over the entire pressure range. Therefore, the cooperative interaction between the 3D ordered tri‐scale graded microstructure and pressure allows the proposed design to effectively enhance the sensitivity and linearity of the piezoresistive pressure sensor across a wide range.

### 2.2 Laser Fabrication and Characterization of the 3D Ordered Tri‐Scale Graded Microstructure

**Figure 2:**
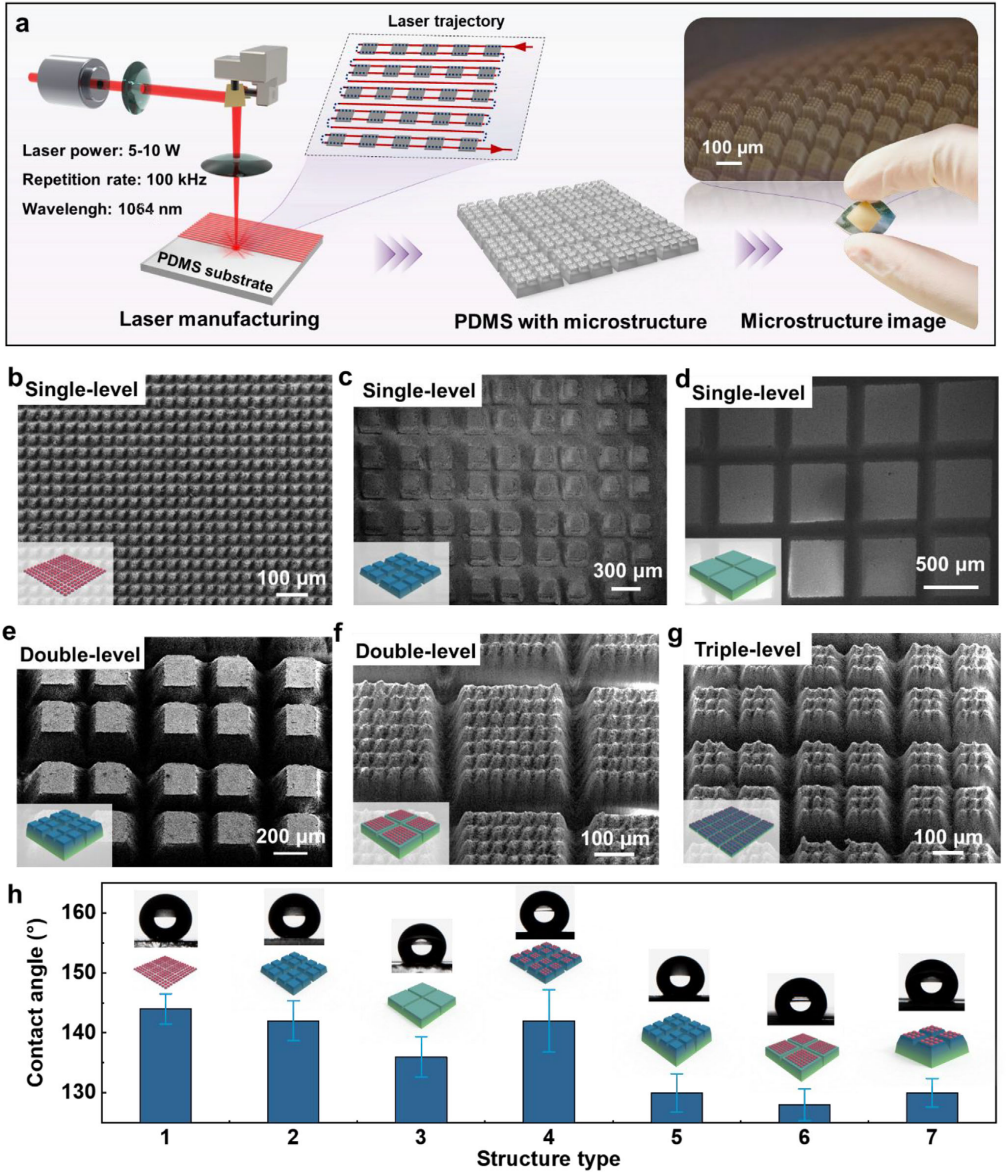
Laser fabrication of 3D ordered graded microstructure. a) Schematic and photo of laser‐fabricated microstructure. SEM images of single‐level microstructure: b) 1#, c) 2#, and d) 3#. SEM images of double‐level microstructure e) 23# and f) 13#. g) SEM image of triple‐level microstructure 123#. h) Hydrophobicity and hydrophilicity characterization of the microstructures. The contact angle decreases gradually as the scale of single‐level microstructure increases from 1# to 3#. The contact angle decreases gradually from secondary to tertiary microstructure. Error bars represent ± standard deviation (SD) from the mean (n = 3).

In this study, a 3D ordered tri‐scale graded microstructure was fabricated using a laser processing technique (**Figure** **2**a). The process employed an infrared picosecond laser with a wavelength of 1064 nm, a pulse width of 10 ps, and a repetition rate of 100 kHz. To generate microstructures at different scales, distinct scanning paths were applied to each grade, combined with cross‐scanning strategies, as shown in Supplementary Figures S3 and S4 (Supporting Information). For every grade, the parameters and number of passes were specifically adjusted.

The quality of microstructure fabrication is determined by the balance between laser energy density and material removal efficiency. When the energy is too low, the laser fails to reach the ablation threshold of PDMS, resulting in adhesion or blurred structures. When the energy is too high, thermal effects become significant, leading to local burning and structural damage. Similarly, an excessively small scanning line spacing causes a high local energy density that leads to melting and adhesion, while an excessively large line spacing results in insufficient energy coverage, leaving some areas unremoved. A slow scanning speed leads to energy accumulation and burning, whereas an overly fast speed results in insufficient energy per spot and incomplete removal. Based on these mechanisms, we systematically investigated the effects of laser power, scanning line spacing, and scanning speed on the fabrication quality of PDMS microstructures. The morphology and dimensions of the fabricated microstructures were observed and analyzed, and optimization was carried out using the most challenging 40 µm‐scale structures as representative cases. The results are shown in Supplementary Figures S5–S7 (Supporting Information). At a laser power of 4.0 W, the energy was insufficient, leading to incomplete removal and adhesion of the structures. At 4.9 W, the energy was excessive, causing structural damage. At 4.6 W, uniform and well‐defined microstructures were achieved. Likewise, an optimized balance between energy density and processing integrity was obtained at a scanning pitch of 0.01 mm and a scanning speed of 2000 mm s^−1^, while other conditions led to adhesion or incomplete removal. Following this optimization procedure, the strategy was further extended to microstructures with scales of 240 and 640 µm, and the final parameters are shown in Supplementary Table S1 (Supporting Information).

In terms of fabrication strategy, a top‐down approach was employed. The smallest 40 µm microstructures were fabricated first, followed by the 240 µm structures. To avoid damaging the pre‐formed 40 µm features, the scanning regions were precisely defined, as shown in Supplementary Figure S3d (Supporting Information). Finally, the largest 640 µm structures were fabricated, with care taken to preserve the integrity of the existing 40 and 240 µm structures. The optical image inset of Figure 2a highlights the well‐defined layered architecture of the resulting microstructures.

Figure 2b–d shows scanning electron microscope (SEM) images of single‐level microstructures with dimensions of 40, 240, and 640 µm. Figure 2e–g displays the corresponding double‐level and triple‐level structures. These images illustrate the dimensional variation across different scales and confirm the precision and reliability of the laser fabrication method.

To further evaluate the surface morphology of the microstructures, contact angle measurements were performed (Figure 2h). For single‐level structures, the contact angle decreased from 143° to 138° as the feature size increased. A comparable trend was observed in double‐level structures, particularly when combined with the 640 µm features, where the contact angle dropped further. The triple‐level structures exhibited a contact angle of ≈130°. These results demonstrate that the fabricated 3D ordered tri‐scale graded microstructures possess distinct hydrophobic properties. Although Samples #1, #6, and #7 with the same minimum structural scale of 40 µm, their hierarchical architectures differ significantly, which governs the apparent wettability. In #1, the surface consists of a continuous array of 40 µm microstructures, which facilitates a stable Cassie‐Baxter state and efficient air entrapment, leading to a large contact angle. In #6, the 40 µm microstructures are distributed only on the 640 µm plateaus, forming a double‐level hierarchy (40–640). The wide spacing between plateaus allows the droplet to bridge across and partially wet the grooves, resulting in a Cassie/Wenzel mixed state and a reduced contact angle. In #7, a triple‐level of 240 µm structures is introduced (40‐240‐640). This further increases the solid‐liquid contact fraction and enhances contact line pinning, while weakening the air‐trapping effect, leading to an even smaller contact angle. These results indicate that the variation in contact angle is predominantly determined by the hierarchical geometry rather than by the minimum feature size alone.

Overall, the characterization confirms that laser processing can reliably produce hierarchical architectures with high structural fidelity, providing a robust basis for optimizing sensor performance in later applications.

### 2.3 Preparation of Pressure Sensing Layer and Sensor Packaging

**Figure 3:**
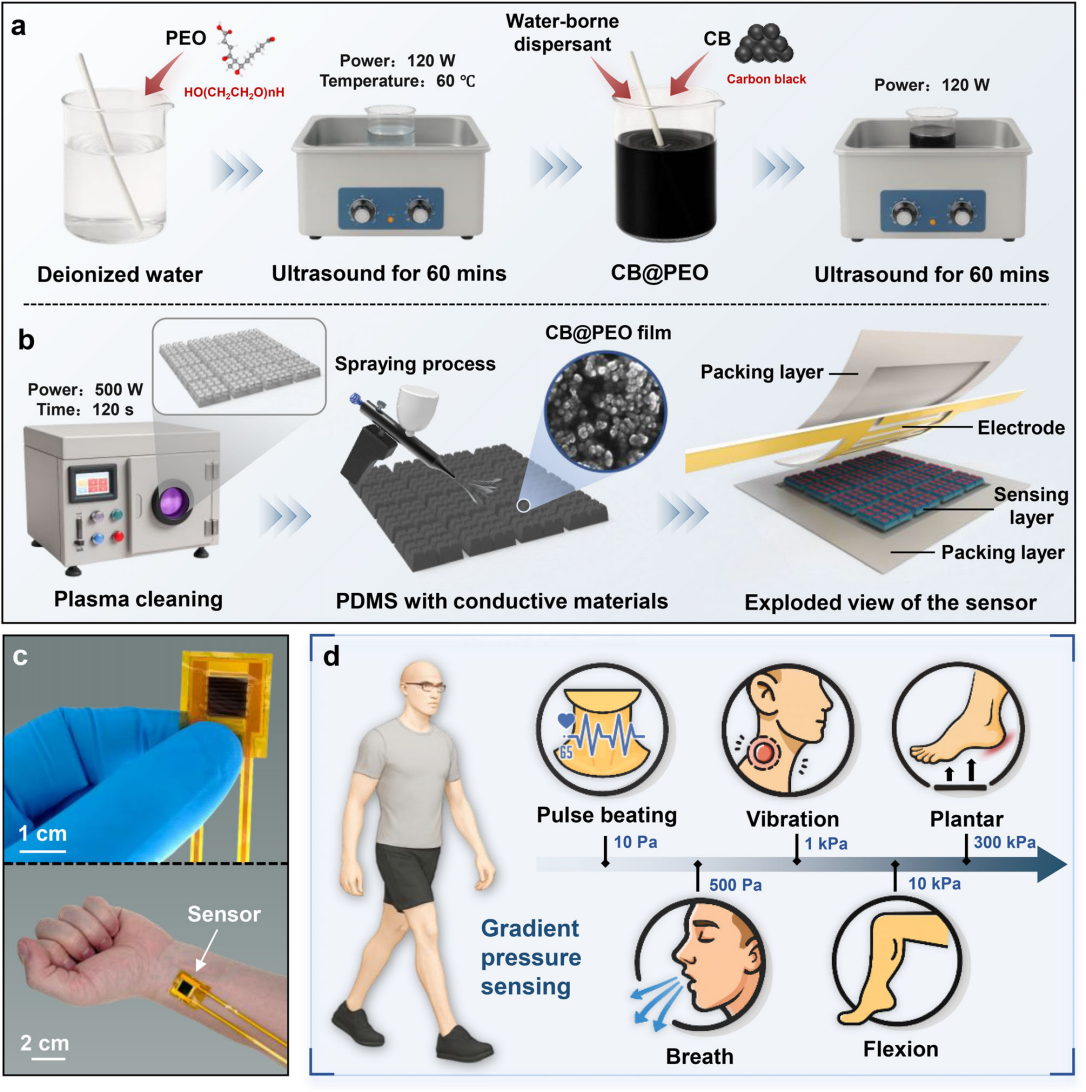
Fabrication of the pressure sensing layer and sensor packaging. a) Process flow for the preparation of the conductive material. b) Spraying of the pressure sensing layer and sensor assembly. c) Image of the pressure sensor and its installation on the wrist for monitoring. d) Physiological health monitoring using the pressure sensor across a wide pressure range from 10 Pa to 300 kPa.

In this study, we selected polydimethylsiloxane (PDMS) as the flexible substrate and used a high‐conductivity mixture of carbon black (CB) and polyethylene oxide (PEO) as the conductive layer, as shown in **Figure** **3**a. PEO is a water‐soluble polymer with thermoplastic properties. When it is thoroughly mixed with a carbon black aqueous solution and undergoes evaporation and curing, a thin film is formed on the surface of the carbon black to prevent its detachment. The dispersant serves to prevent the carbon black particles from settling and agglomerating.^[^ ^37^ ^]^ First, PEO powder was dissolved in deionized water. Then, conductive CB was added to the solution along with a non‐ionic surfactant to improve CB dispersion. The mixture was sonicated to form a homogeneous CB@PEO solution. Detailed preparation procedures are provided in the Experimental Section. The CB@PEO material prepared through ultrasonic dispersion can be stored for an extended period. The solution we prepared remained well‐dispersed even after six months of storage, without any occurrence of phase separation between water and conductive materials, indicating that no aggregation of CB@PEO, as shown in Supplementary Figure S8 (Supporting Information).

The prepared CB@PEO solution was then uniformly deposited onto the surface of the 3D ordered tri‐scale graded microstructure using a spraying technique (Figure 3b). Considering that the contact angle of the 3D ordered graded geometric microstructure surface was greater than 130°, indicating hydrophobicity, we first treated the PDMS substrate with oxygen plasma cleaning (JL‐V05, JINLAI Technology Co., Ltd, China) to improve its hydrophilicity. In the plasma, high‐energy particles interact with the PDMS surface, introducing polar groups (such as hydroxyl and carboxyl groups) to increase surface polarity and enhance hydrophilicity. The plasma cleaning power was set to 500 W, with a cleaning time of 120 s. After oxygen plasma treatment, the hydrophilicity of the PDMS surface was significantly improved, providing better adhesion for subsequent coating deposition. During the spraying process, the PDMS substrate was placed on a heated stage at 80°C to ensure the formation of a uniform and dense pressure sensing film. To ensure the uniform deposition of CB@PEO materials, we quantitatively controlled the solution volume for each pressure‐sensing layer, fixing the deposition volume at 0.25 mL. Since the spraying solution was dispersed in the form of a mist at a fixed angle, the spraying area could be regulated by adjusting the spraying height, thereby achieving quantitative and uniform deposition. Thanks to the quantitative spraying process, the 8 different pressure sensing layers exhibit stable initial resistance in the range of 50–60 MΩ (Figure S9, Supporting Information), with only minor variations. The thickness of the deposited film was ≈5.8 µm (Figure S10, Supporting Information). An EDS analysis of the CB@PEO elements was performed, and the results are shown in Supplementary Figure S11 (Supporting Information). The main components of CB@PEO are carbon and oxygen, with carbon accounting for 94.48% and oxygen for 5.27%.

Finally, the sensor was packaged in a sandwich configuration, where the pressure sensing layer and interdigital electrode were assembled face‐to‐face. PI tape was applied to securely hold the two layers together (Figure 3b, rightmost). The completed device is shown in Figure 3c, including a photo of the sensor and its attachment to the wrist. The sensor was then tested for physiological signal detection, covering pulse, respiration, throat vibration, joint bending, and foot pressure (Figure 3d). With this design, our sensor is capable of providing precise pressure detection under different physiological conditions, offering a reliable solution for health monitoring and human‐machine interaction.

### 2.4 Performance of Pressure Sensing

**Figure 4:**
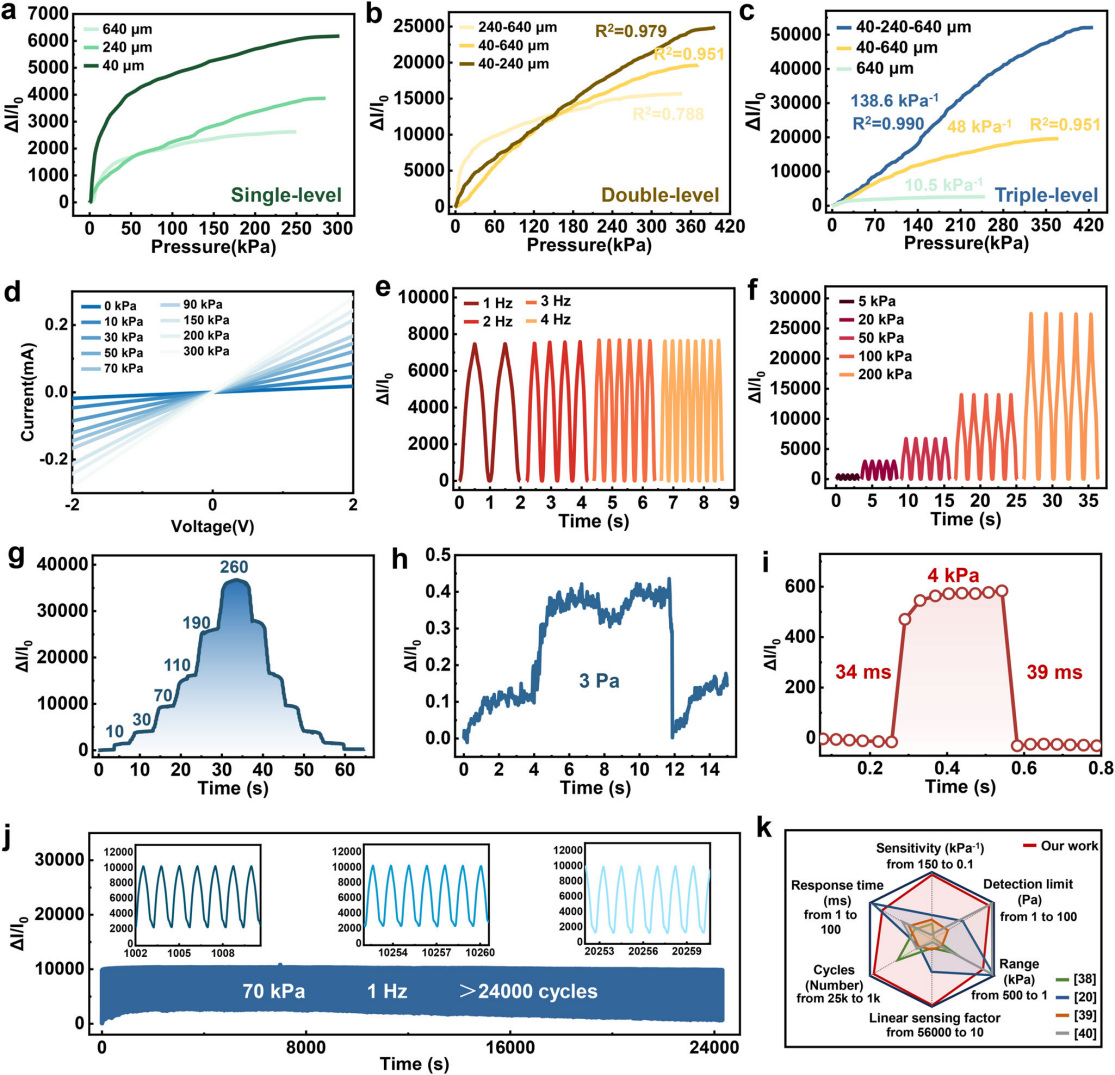
Sensing performance of the 3D‐OTGM pressure sensor. a) The relative current change as a function of pressure of piezoresistive sensors with a single‐level microstructure array. b) The relative current change as a function of pressure of piezoresistive sensors with a double‐level microstructure array. c) The relative current change as a function of pressure of piezoresistive sensors with a triple‐level microstructure array. The linear sensing factor of the 3D‐OTGM pressure sensor reaches 55 440. d) Linear relation of the *I–V* curves of the sensor at different pressures. e) Time‐dependent variation of current at different vibration frequencies. f) The current response to cyclic pressures of 5, 20, 50, 100, and 200 kPa. g) A stepwise pressure increasing from 0 to 260 kPa detected by the 3D‐OTGM sensor. h) The 3D‐OTGM sensor detection limit. i) Dynamic response of the 3D‐OTGM sensor. j) Stability of the 3D‐OTGM under a cyclic pressure with an amplitude of 70 kPa and frequency of 1 Hz over 24 000 cycles. k) Comparison of the sensing performance of the 3D‐OTGM sensor with reported high‐performance microstructure sensors.

To validate the effectiveness of the proposed design, we systematically characterized the performance differences of sensors with different graded geometric microstructure types, including sensitivity, linearity, and sensing range. The sensitivity (*S*) of the pressure sensor is defined as:

(3) $$S=\frac{\delta \left(\Delta I/I_{0}\right)}{\delta P}$$

where Δ*I* is the change in current under different applied pressures (*P*), and *I*_0_ is the initial current before pressure is applied. **Figure** **4**a shows the pressure‐current characteristic curve for the single‐level microstructure sensor. As the microstructure size decreases, the sensitivity of the sensor significantly increases within the 0–25 kPa range; however, at 50 kPa, the sensitivity sharply drops, exhibiting noticeable nonlinear behavior. Figure 4b presents the pressure‐current characteristic curve for the double‐level microstructure sensor, which shows that this sensor has a wider range compared to the single‐level microstructure sensor. The #12 (40‐240 µm) structure sensor demonstrates the highest sensitivity and exhibits good linearity, with an *R* ^2^ value of 0.979.

Figure 4c shows the performance of 3D ordered tri‐scale graded microstructure array sensor (triple‐level, denoted as 3D‐OTGM) compared with the double‐level (#13) and single‐level (#3) microstructure sensors. The 3D‐OTGM sensor shows a significant increase in sensitivity, reaching 138.6 kPa^−1^ and demonstrates excellent linearity (*R* ^2^ = 0.99). Its linear sensing range also extends up to 400 kPa, outperforming both the double‐level and single‐level microstructure sensors. This result further verifies the significant performance advantages of the 3D‐OTGM sensor based on its design principles. We performed four reproducibility tests of the sensor, and the results indicated good repeatability overall, with the four curves closely overlapping across most of the pressure range (Figure S12, Supporting Information). We quantitatively evaluated the maximum difference among the experiments: at a pressure of ≈150 kPa, the largest deviation was observed, where the outputs of two curves were 16,000 and 23,500 (Δ*I*/*I*_0_), yielding a difference of ≈7 500. Relative to the full scale of the sensor (FS ≈ 55,000), this deviation was ≈13.9%. Overall, the reproducibility tests confirmed that the sensor demonstrates stable performance across most of its operating range.

Figure 4d shows the current‐voltage (IV) curves of the sensor at different pressures. The IV curve intersects the origin and increases monotonically in a linear fashion, indicating that the sensing layer exhibits ohmic contact characteristics and stable electrical performance. To study the electrical response of the 3D‐OTGM sensor at different frequencies, we applied a 10 kPa pressure at frequencies of 0.5 Hz, 1 Hz, 2 Hz, and 4 Hz. The 3D‐OTGM sensor displayed stable responses, eliminating frequency dependence, as shown in Figure 4e. Figure 4f shows the relative change in current under dynamic loading at different pressures, with stable and repeatable responses observed over five cycles. The 3D‐OTGM sensor demonstrated a monotonic increase in current during stepwise loading (Figure 4g). The key performance metrics for the pressure sensor include minimum detectable pressure, response time, and recovery time. The sensor can detect pressures as low as 3 Pa, with a response time of 34 ms and a recovery time of 39 ms, as shown in Figure 4h–i. The minimum detection limit under high pressure was tested, and the results are shown in Supplementary Figure S13 (Supporting Information). The minimum detectable pressures under preloads of 1 kPa, 10 kPa, and 100 kPa are 7 Pa, 15 Pa, and 70 Pa, respectively. When normalized to the full‐scale range of 400 kPa, these correspond to 0.00175 %FS, 0.00375 %FS, and 0.0175 %FS, respectively. Although the absolute resolution degrades with increasing preload, the values remain below 0.02 %FS, demonstrating that the sensor maintains high resolution across the entire 0–400 kPa range.

Notably, the 3D‐OTGM sensor exhibits excellent stability over prolonged use, withstanding 24 000 cycles of 70 kPa pressure at a frequency of 1 Hz, with no noticeable output drift (Figure 4j). However, the baseline drift shows an initial increase followed by a gradual decrease. The baseline response variation can be attributed to the structural evolution of the conductive network and the interface during repeated loading/unloading. At the initial stage of cycling, the viscoelastic deformation of the PDMS substrate induces residual strain, and slight interfacial sliding occurs, resulting in baseline drift. With further cycling, the PDMS substrate gradually releases internal stress and the electrode‐sensing layer contact becomes more compact, leading to a gradual recovery of the baseline. This phenomenon represents a typical “training‐stabilization” process of resistive‐type flexible pressure sensors under long‐term cyclic testing. In addition, during testing, the sensor was fixed onto an I‐beam load cell using adhesive tape (Figure S14, Supporting Information), and prolonged cyclic pressing may have caused slight sliding of the underlying load cell, which can also contribute to baseline drift. It should be emphasized that the response amplitude remained nearly unchanged throughout the cycling process, indicating that the functional stability and durability of the device were not affected. We compared the performance of the 3D‐OTGM sensor with existing microstructure piezoresistive sensors, as shown in Figure 4k. The comparison demonstrates significant advantages of our sensor in terms of sensitivity, linear range, response time, and cyclic stability.^[^ ^20^, ^38^, ^39^, ^40^ ^]^ The linear sensing factor (LSF), the product of the sensitivity and the linear sensing range,^[^ ^19^ ^]^ of our 3D‐OTGM sensor reaches 55 440, which overweighs the values of any pressure sensors found in the literature.^[^ ^4^, ^5^, ^6^, ^7^, ^8^, ^9^, ^10^, ^11^, ^20^, ^38^, ^39^, ^40^ ^]^

### 2.5 Physiological Signal Health Monitoring

The 3D‐OTGM sensor demonstrates high sensitivity, a broad linear response range, rapid response, and long‐term stability, enabling reliable detection of subtle physiological signals. In this section, we systematically assess its performance in monitoring human pulse (carotid artery, jugular vein, and wrist), respiration, throat vibrations, joint bending, and plantar pressure.

**Figure 5:**
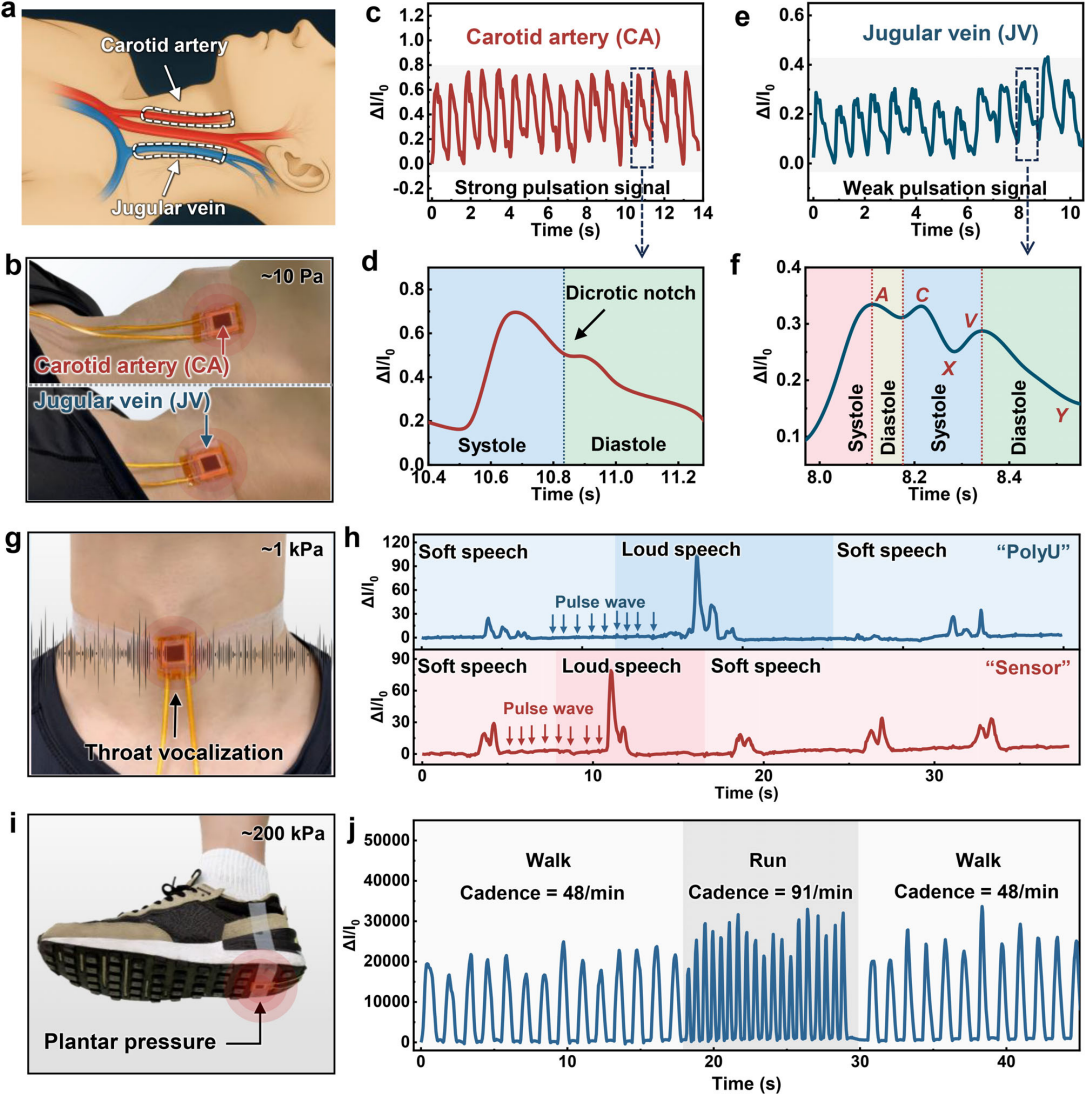
3D‐OTGM sensor health detection. a) Schematic of the positions of the carotid artery (CA) and jugular vein (JV) on the human neck. b) Photo of the sensor installed on the neck. c) Monitoring of carotid artery signals. d) Enlarged view of the carotid artery waveform. e) Monitoring of jugular vein signals. f) Enlarged view of the jugular vein waveform. g) Photo of the sensor installed on the throat. h) Current response generated during the speech of “PolyU” and “Sensor”. i) Photo of the sensor installed on the shoe sole. j) Foot pressure changes during slow walking and running.

**Figure** **5**a shows the schematic of the carotid artery and jugular vein locations on the human body. We affixed the sensor to the subject's neck to non‐invasively measure and monitor the central blood pressure waveform and pulse of the carotid artery (CA) and jugular vein (JV), as shown in Figure 5b. The central blood pressure waveform provides valuable information regarding cardiovascular diseases or events.^[^ ^41^, ^42^ ^]^ The CA, located ≈2.5 cm beneath the skin, is responsible for pumping rich blood from the left ventricle and left atrium to the rest of the body, producing a strong pressure waveform signal that is closely related to left heart activity.^[^ ^42^, ^43^ ^]^ In contrast, the JV, located deeper in the neck, produces a much weaker pulse waveform signal. In clinical practice, continuously and accurately monitoring the JV pulse waveform in real‐time is a highly challenging task.

Figure 5c shows the continuous waveform sequence of the CA pulse, which exhibits strong pulsatile signals recorded by the sensor. Figure 5d shows an enlarged view of the CA waveform, revealing the detailed characteristic peaks and shapes of the vascular pulsations. The characteristic CA pulse waveform consists of three main components: the systolic phase, the dicrotic notch, and the diastolic phase. These components provide valuable clinical information related to left heart activity.^[^ ^43^ ^]^ We place the sensor on the neck vein area for testing (Figure 5e), showing the continuous waveform sequence of the JV pulse with weaker pulsatile signals. Figure 5f shows an enlarged view of the JV pressure pulsation waveform, which features a biphasic pattern with three peaks: the A peak (right atrial contraction), C peak (right ventricular contraction, causing the tricuspid valve to bulge into the right atrium), and V peak (venous filling and increased venous pressure), as well as two troughs: the X trough (right ventricular contraction, atrial relaxation) and Y trough (tricuspid valve opening and right ventricular filling) ^[^ ^43^, ^44^ ^]^ The recorded pulse waveforms exhibited distinct differences CA pulse waveform. The JV signal is much weaker in terms of pressure intensity compared to the CA signal. In addition to carotid pulse signal detection, our 3D‐OTGM sensor can also monitor wrist pulse signals (Figure S15, Supporting Information). The stable, uniform, and recognizable waveform signals indicate that our tri‐scale graded microstructure sensor can continuously and accurately monitor cardiovascular events in a non‐invasive manner.

Figure 5g shows the results of throat vibration monitoring. The test subject pronounced the words “PolyU” and “Sensor” in both soft and loud voices. The vibration of the throat caused by speech compression deforms the sensor, allowing it to detect pressure changes induced by the vibrations. Figure 5h shows the changes in the electrical signal of the sensor during speech. When saying “PolyU”, three characteristic peaks of fluctuation can be clearly observed. When saying “Sensor”, the signal shows higher peaks at both ends and a lower peak in the middle. The characteristic peak waveform signals produced by both soft and loud speech are generally consistent. Interestingly, even without speech, due to the bandage binding the sensor to the neck, the neck sensor is still able to detect weak vibrations generated by the carotid pulse (Figure S16, Supporting Information), demonstrating the high precision in detecting subtle signals of the 3D‐OTGM sensor.

Figure 5i shows the application of the sensor installed on the foot for foot pressure detection. During normal walking, the walking frequency is ≈50 steps per minute, while during fast walking, the walking frequency is ≈100 steps per minute. Figure 5j shows gait detection during slow walking and running, where the foot pressure generated by the gait is ≈200 kPa. The walking frequency for slow walking is 48 steps per minute, and for running, it is 91 steps per minute. Thanks to the fast response speed, the sensor successfully detects changes in foot pressure at different walking frequencies. Furthermore, the sensor can effectively monitor finger bending (Figure S17, Supporting Information), with good response to different finger bending angles, making it suitable for recognizing most hand gestures. At the same time, the sensor can also be used for respiration monitoring (Figure S18).

### 2.6 Flexible Human‐Machine Interactions

Flexible pressure sensors exhibit excellent wearability and comfort, allowing them to contact the human body without causing harm. As a result, they are widely used in body data acquisition. Additionally, compared to traditional human‐machine interaction methods, flexible pressure sensors can more naturally perceive the user's movements and status, enhancing the accuracy and convenience of the interaction. Therefore, by integrating flexible pressure sensors into the human‐machine interface of robots, signals acquired from wearable devices can drive smart robots, enabling more natural interactions.

**Figure 6:**
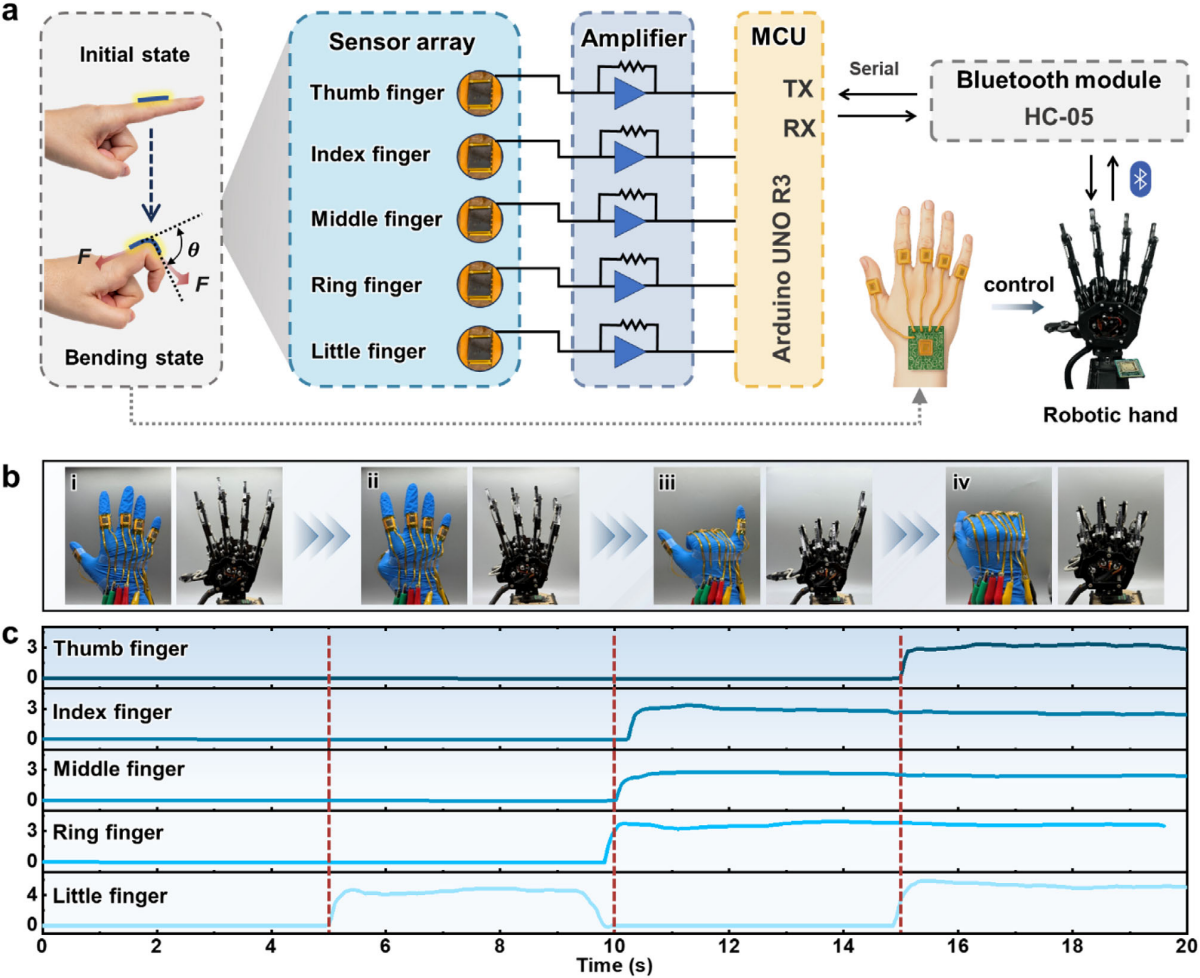
The 3D‐OTGM pressure sensor for remote control of a robotic hand. a) System‐level block diagram of the robotic control system; the baud rate of the HC‐05 Bluetooth module was set to 9600. b) Sensor array controlling the five fingers of the robotic hand. c) Variation in the output signals collected by the sensor array.

**Figure** **6**a shows the schematic of the human‐machine interaction system framework. In this system, wireless data acquisition technology is employed to collect real‐time data, enabling the capture of hand movement sensory data. As shown in Figure S17 (Supporting Information), the sensor can accurately detect changes in finger bending. Based on this, we designed and created five sensors, which are integrated into a sensor array in the glove. The sensors are installed at the second joint of the fingers of a nitrile glove to create a sensor glove. The system consists of an operational amplifier, an Arduino development board, a Bluetooth module (HC‐05), and a robotic hand. When the fingers bend, the electrical signal generated by the sensor is amplified and filtered by the operational amplifier. The output analog signal is then converted into a digital signal via pulse width modulation, which controls the corresponding finger bending movement of the robotic hand.

Figure 6b shows the test subject performing the “5”, “4”, “6”, and “0” gestures, with the robotic hand performing the corresponding actions. The 3D‐OTGM sensors collect pressure signals from the five finger joints, reflecting the real‐time movement state of the fingers. When the fingers remain stationary, the signals from each finger are stable, preventing abnormal vibrations in the robotic hand. When the fingers bend and the output electrical signal exceeds 1.5 V, the system detects finger bending, and the corresponding joint of the robotic hand executes the bending motion, as shown in Figure 6c. We conducted a quantitative analysis of the delay, the baud rate of the HC‐05 Bluetooth module was set to 9600. By recording the sensor signal and the time at which the upper computer received the signal using a serial port assistant, the average delay was measured to be ≈140 ms. This delay mainly originated from the sampling cycle delay (delay(100)) in the wireless transmission program as well as the inherent latency of the Bluetooth protocol. Regarding accuracy, we conducted 20 tests of hand opening and closing actions. Since the control logic was based on a simple binary threshold judgment (0/1), the actions of the robotic hand were fully consistent with the commands, achieving an accuracy close to 100%. This robotic control system has vast application potential, especially in medical and military fields, where it can be used for remotely performing dangerous tasks.

In addition to driving robotic gestures, considering the programmability of the human‐machine interface, the pressure threshold of the wireless wearable glove can be reasonably set according to different functional requirements. For example, the human‐machine interface can be used as a switch to control the responses of the thumb, index finger, middle finger, and little finger. When the pressure‐induced current response exceeds the set threshold, it can manipulate a virtual container to perform specified actions. Another method is to use multi‐threshold settings to expand the interaction functions, such as achieving multifunctional interaction across different pressure ranges from light to heavy.

## 3 Conclusion

Traditional flexible pressure sensors face significant challenges in simultaneously achieving high sensitivity and a wide linear range. In this study, we propose a flexible pressure sensor with a 3D ordered tri‐scale graded microstructure and fabricated using laser processing, achieving a continuously deformable contact interface, which significantly reduces the common compression hardening effect. This, in turn, allows us to extend the linear response range of the sensor while maintaining high sensitivity. The sensor we developed demonstrates ultra‐high sensitivity (138.6 kPa^−1^), excellent linearity (*R* ^2^ = 0.99) over a wide measurement range (up to 400 kPa), and outstanding mechanical durability (capable of withstanding 24 000 cycles). Additionally, the 3D‐OTGM sensor demonstrates strong potential for medical monitoring of physiological signals and for haptic sensing in human‐machine interaction. This study validates the effectiveness of the tri‐scale graded microstructure design and offers new perspectives for advancing flexible sensing technologies. Through laser processing, we establish a scalable and effective fabrication strategy with broad applicability. Despite these advantages, laser processing still faces challenges for large‐scale manufacturing, such as relatively long processing time, high equipment cost, and limitations in throughput. In the future, combining advanced laser techniques with other scalable methods (e.g., printing or roll‐to‐roll processing) may further improve fabrication efficiency and enable practical mass production. Overall, these findings provide a foundation for future wearable diagnostic platforms and smart biomedical systems, and they will help drive flexible sensor‐based health monitoring and human‐machine interaction technologies toward greater precision and wider adoption.

## 4 Experimental Section

### Laser Fabrication of 3D Ordered Tri‐Scale Graded Microstructure

By adjusting the laser processing parameters, the desired microstructures can be customized. A picosecond infrared laser (1064 nm, PINE 1064‐20, China) was used to pattern PDMS substrates. The processing parameters of the laser system were summarized in Table S1 (Supporting Information). Microstructure geometry was controlled by adjusting laser power, scanning speed, repetition rate, and the number of passes, enabling the fabrication of tri‐scale graded architectures with high structural fidelity.

### Preparation CB@PEO Conducting Solution

First, 0.3 g of polyethylene oxide (PEO, Mv ≈300 000) powder was dissolved in 50 mL of deionized water and heated in a 60°C water bath while stirring and sonicated for 1 h to ensure complete dissolution. Next, 1 g of conductive carbon black (CB) (XFI15, Nanjing/Jiangsu XFNANO Materials Tech Co., Ltd, China) was added to the PEO solution. Simultaneously, 1.2 g of a non‐ionic surfactant containing aromatic groups was added as a dispersing agent to improve the uniform dispersion of CB. The CB and PEO mixture was then placed in a 120 W ultrasonic cleaner for 1 h to disperse, forming the CB@PEO solution.

### Conductive Material Spraying

The static contact angle of the laser‐fabricated microstructure array was greater than 130° (Figure 2h). To improve surface hydrophilicity, the samples were treated with oxygen plasma (500 W, 120 s). Conductive material was then deposited by pneumatic spraying onto the PDMS array placed on a heated stage (80 °C), forming a uniform and dense sensing layer.

### Fabrication of Electrode and Packaging of Sensor

Polyethylene terephthalate (PET) films were cleaned with deionized water and dried. A 20 nm titanium adhesion layer and a 30 nm gold conductive layer were deposited onto the PET substrate via magnetron sputtering (EXPLORER‐14, USA). The gold layer was patterned using a 355 nm ultraviolet laser (JPT, SEAL‐355‐10S, China), with the following parameters for the removal of gold: single scan, scanning speed of 800 mm s^−1^, frequency of 27 kHz, and 0.1 mm hatch spacing. Subsequently, laser cutting of individual electrodes was performed using optimized parameters: single scan, speed of 75 mm s^−1^, frequency of 33 kHz, and 0.1 mm scan spacing. Finally, the sensor was packaged in a “sandwich” structure and assembled with a 50 µm thick polyimide (PI) encapsulation tape.

### Human‐Machine Interaction System

The arm and gripper movements were coordinated via a sensor‐guided. An Arduino UNO R3 served as the main controller, with wireless communication supported by an HC‐03 Bluetooth module.

### Characterizations and Measurements

A scanning electron microscope (Zeiss Corporation, SUPRA55 SAPPHIRE, Germany), equipped with an energy dispersive spectrometer (EDS), was used to characterize the structure and morphology of the microstructure. The pressure applied to the sensors during tests was controlled by a motorized motion platform (FUYU, FLS40, China) with a motion controller (FUYU, FSC‐2A, China). Pressure acquisition was done via a parallel beam pressure transducer (HY, HYPX‐017, China). The electrical resistance of the sensor was measured using a digital source meter (Keithley, DAQ6510, USA). The IV curve of the sensor was measured using a digital source meter (Keithley 2400, USA). The contact angles of the microstructure array were measured using a contact angle analyzer (Bangyi, JY‐PHa, China). Oxygen plasma treatment (JINLAI, JL‐V05, China) was employed to modulate the surface wettability of the microstructures.

### Human Research Participants

A male aged 31 participated in the physiological signals monitoring study. The purposes and significances of the experiment were informed to the participant before the survey. Participants provided informed consent before the experiment.

### Ethics Statement

All procedures during the testing of human participants were approved by the Medical Ethics Committee of Xiamen University. The informed consent of all participants was obtained prior to inclusion in this study.

## Conflict of Interest

The authors declare no conflict of interest.

## Supporting information



Supporting Information

## Data Availability

The data that support the findings of this study are available from the corresponding author upon reasonable request.
