# Supplementary material for: Ultra‐Sensitive and Linear Flexible Pressure Sensors with Tri‐Scale Graded Microstructures for Advanced Health Monitoring and Robotic Perception
Source: Adv Sci (Weinh). 2025 Oct 20;13(1):e16810. doi: 10.1002/advs.202516810 (PMC12767126; doi:10.1002/advs.202516810)
Supplement: Supplementary file 1 — Supporting Information [file ADVS-13-e16810-s001.docx]

Supporting Information

**Ultra-Sensitive and Linear Flexible Pressure Sensors with** **Tri-Scale Graded Microstructures for Advanced Health Monitoring and Robotic Perception**

Rui Chen^1^, Chi Fai Cheung^1^*, Qixian Zhang^1^, Tao Luo^2^, Rui Gao^1^, Wei Zhou^2^*, Chunjin Wang^1^*

^1^ State Key Laboratory of Ultra-Precision Machining Technology, Department of Industrial and Systems Engineering, The Hong Kong Polytechnic University, Kowloon, Hong Kong, China

^2^ Pen-Tung Sah Institute of Micro-Nano Science and Technology, Xiamen University, Xiamen, 361102, China

* Corresponding author. Email: chunjin.wang@polyu.edu.hk; weizhou@xmu.edu.cn; benny.cheung@polyu.edu.hk

Figure S1. Resistance of interdigitated electrodes.

Figure S2. Initial resistance of CB@PEO pressure sensing layer.


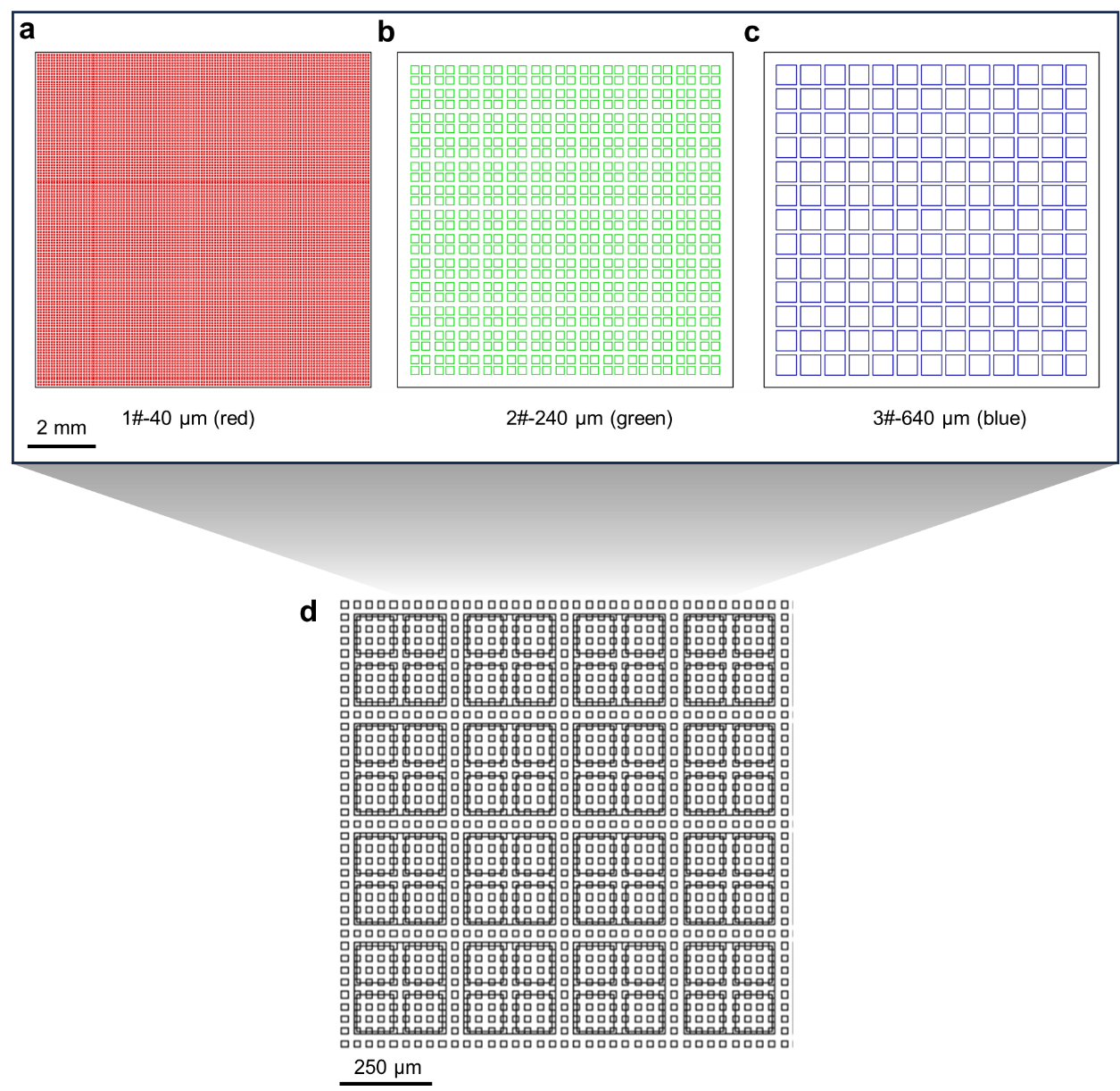


Figure S3. Scanning area segmentation for the laser process, divided into three grades and fabricated using three different sets of laser parameters. a) 40 μm scale. b) 240 μm scale. c) 640 μm scale. d) overall processing trajectory of the three scale combinations.


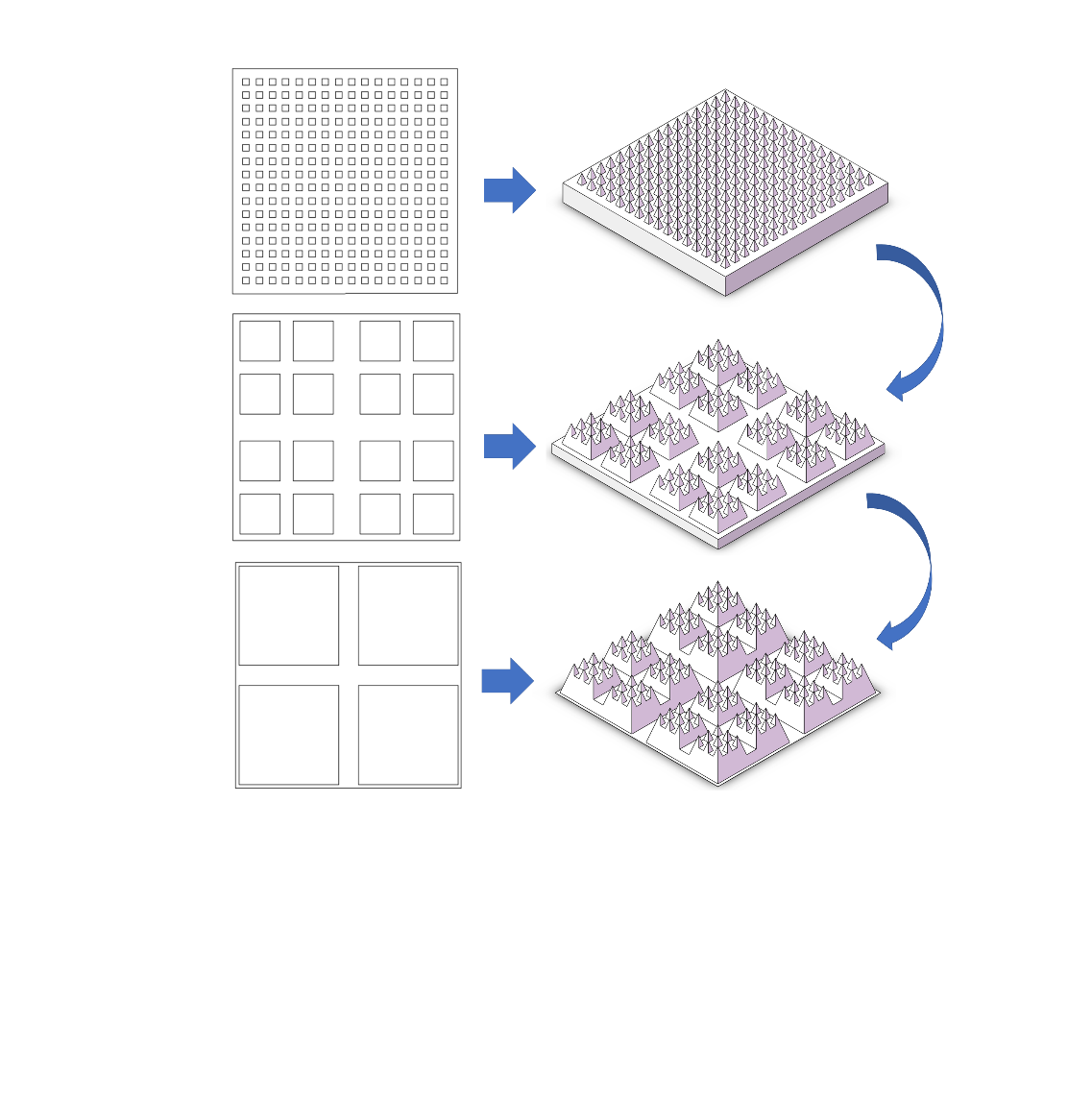


Figure S4. Schematic diagram of tri-scale graded microstructures processing.


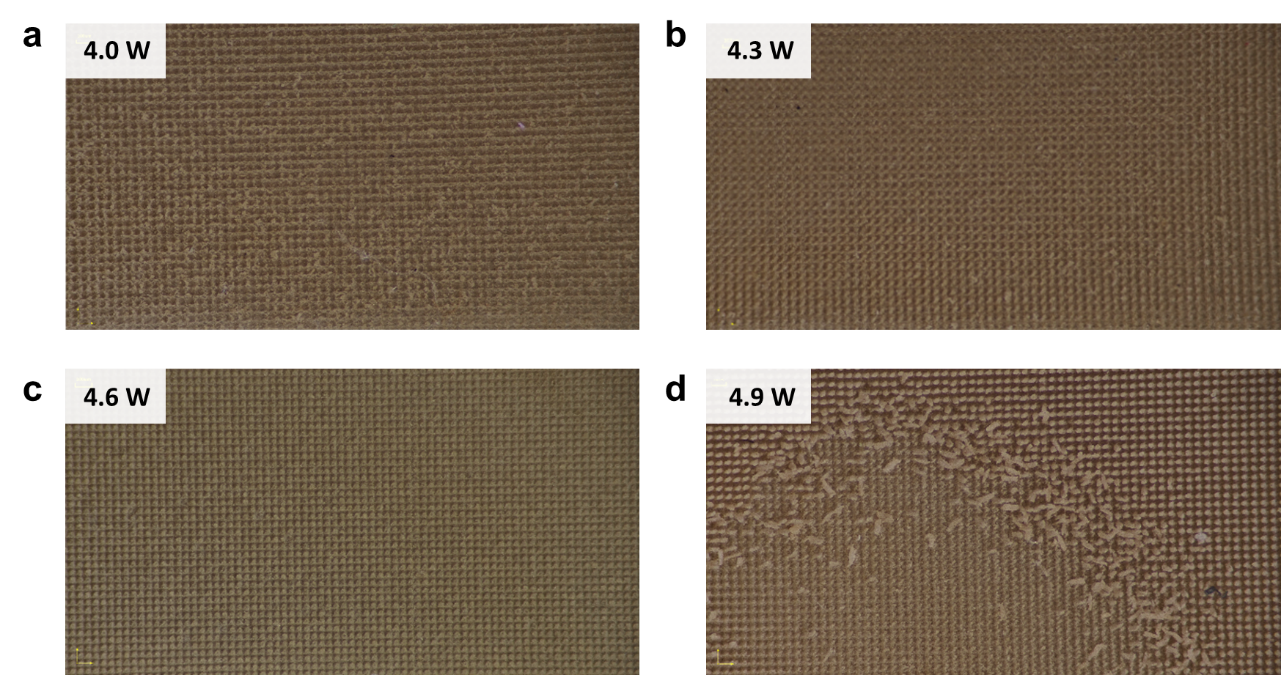


Figure S5. Effect of laser power on PDMS material removal. Power: a) 4.0 W, b) 4.5 W, c) 4.6 W and d) 4.9 W.


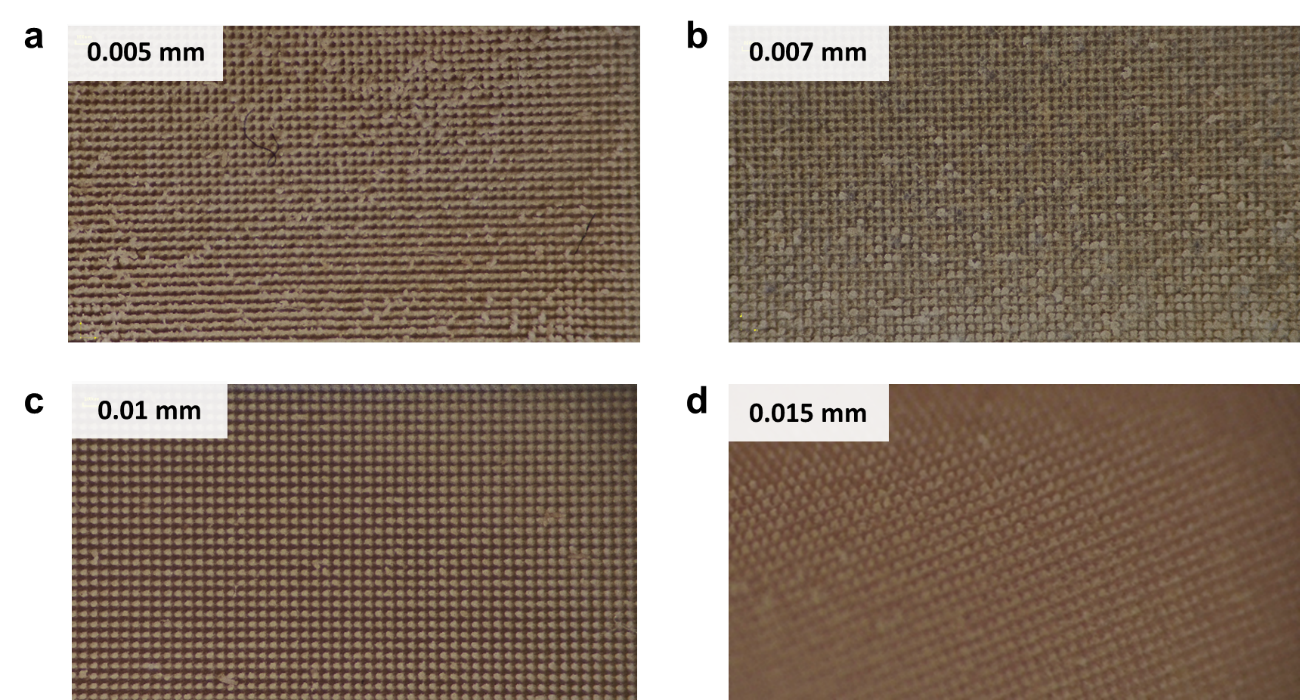


Figure S6. Effect of scanning line spacing on PDMS material removal. Scanning line spacing: a) 0.005 mm, b) 0.007 mm, c) 0.01 mm and d) 0.015 mm.


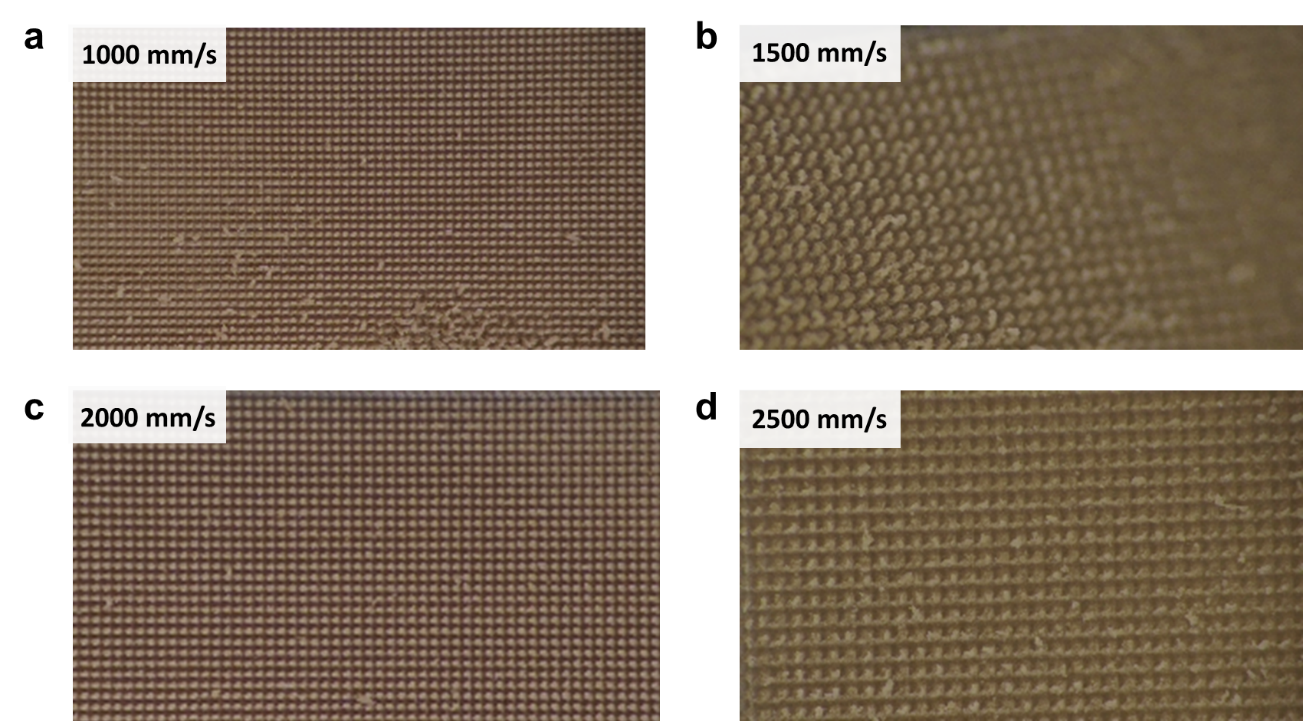


Figure S7. Effect of scanning speed on PDMS material removal. Scanning speed: a) 1000 mm/s, b) 1500 mm/s, c) 2000 mm/s and d) 2500 mm/s.


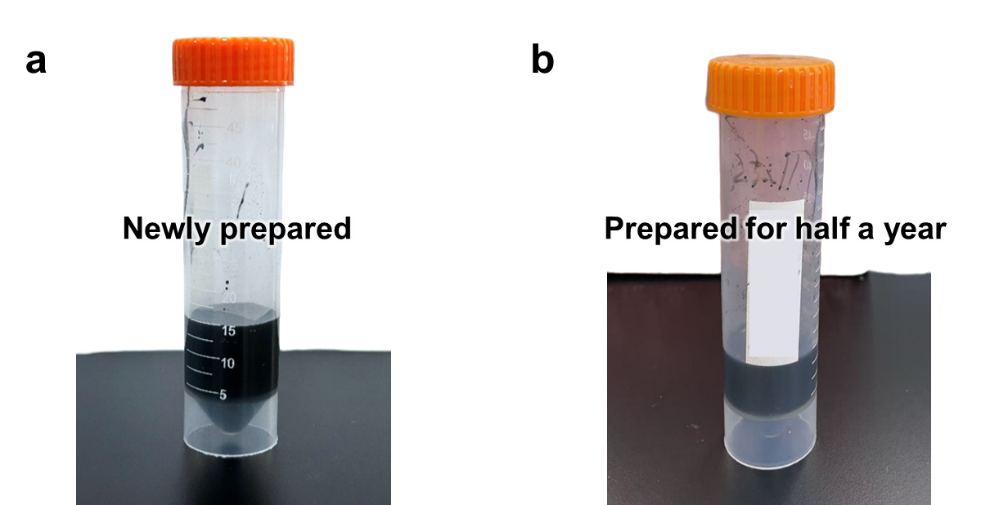


Figure S8. Dispersibility of CB@PEO solution. a) Newly prepared CB@PEO solution. b) CB@PEO solution stored for half a year.

Figure S9. Initial resistance measurement results of different 8 samples.


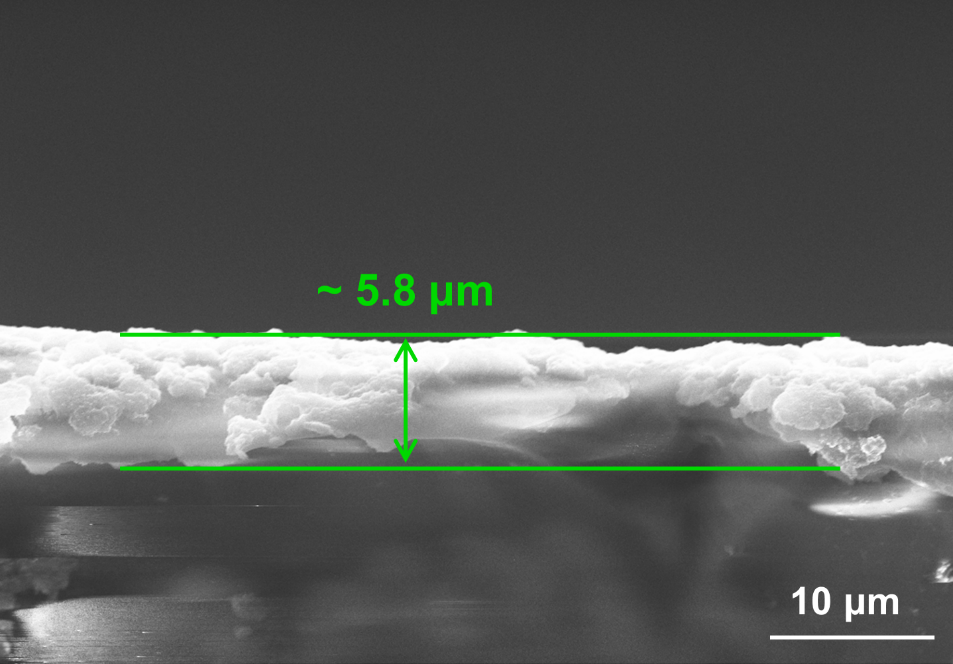


Figure S10. SEM image of CB@PEO film showing a thickness of approximately 5.8 μm.


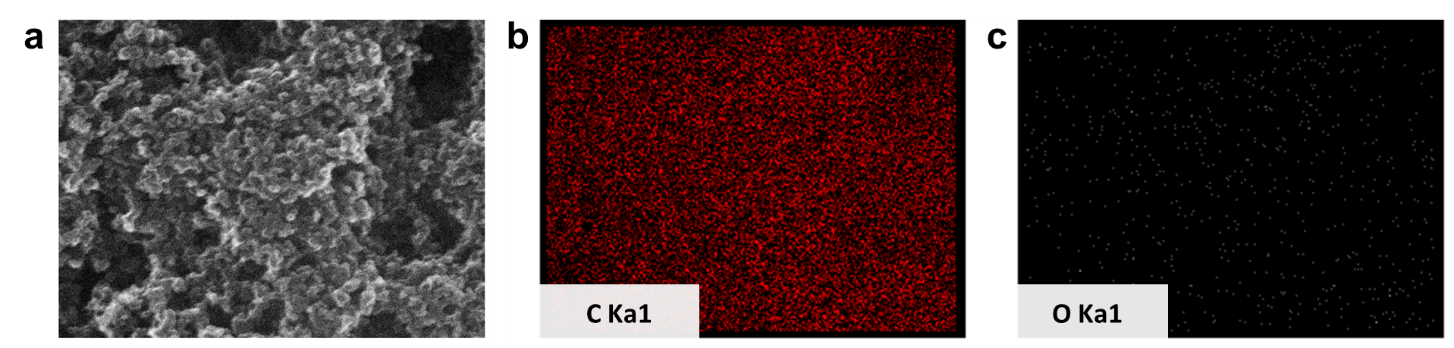


Figure S11. EDS analysis of CB@PEO conductive film. a) The CB@PEO film SEM image. Distribution of b) oxygen and c) C elements in CB@PEO.


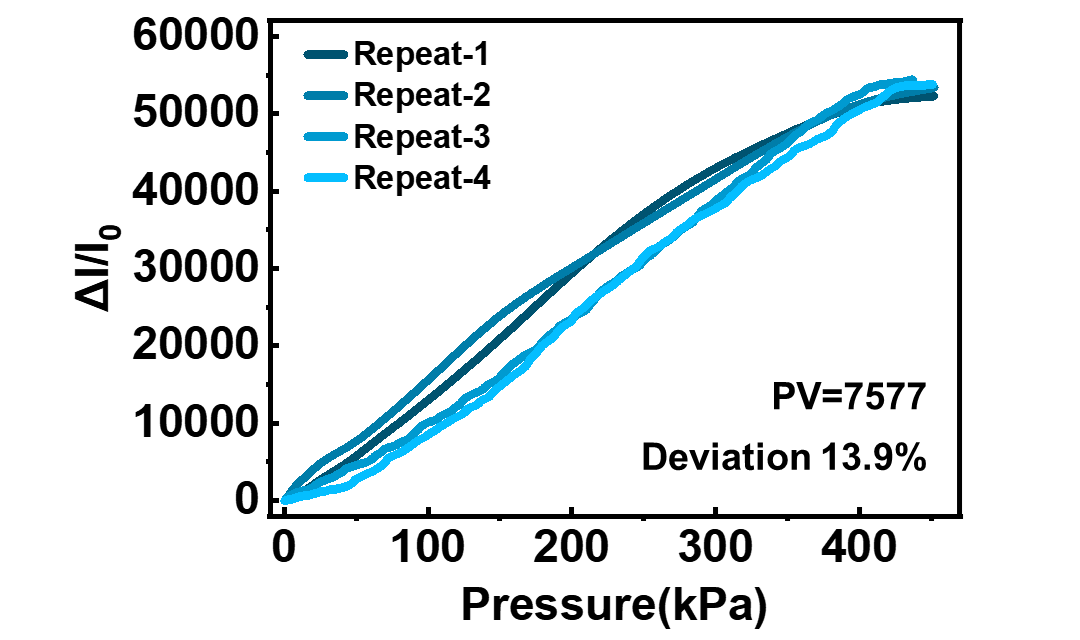


Figure S12. Repeatability test of 3D-OTGM sensors.


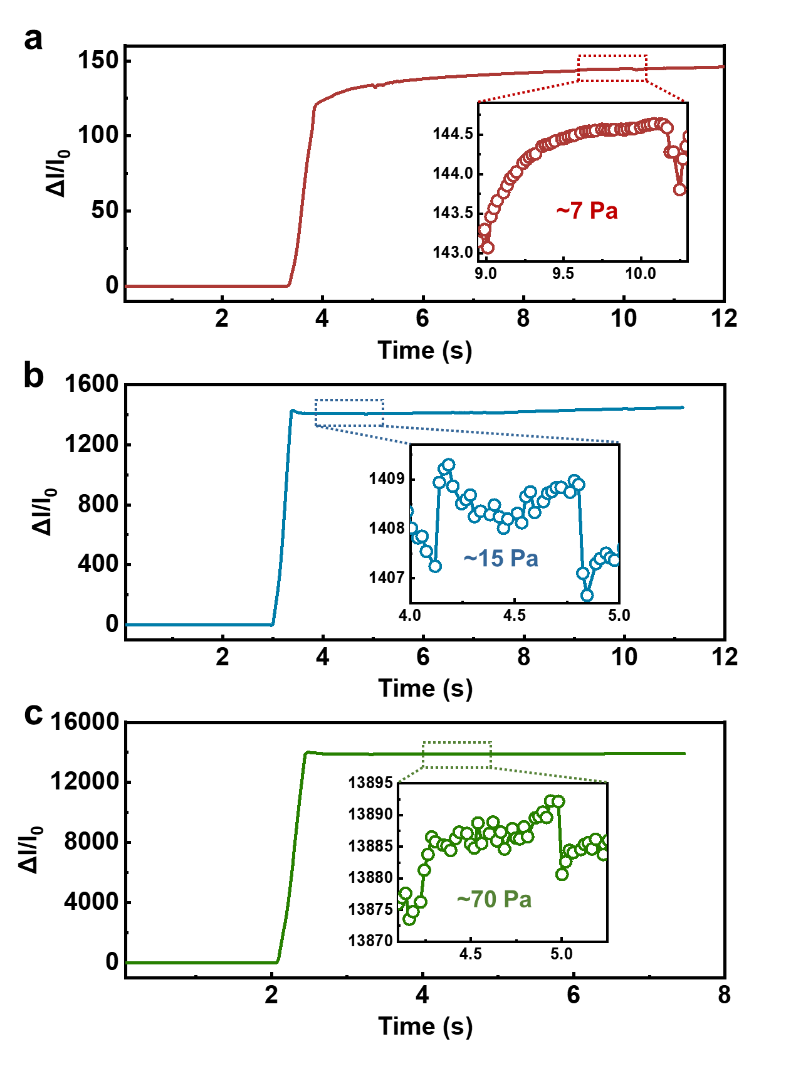


Figure S13. Sensor detection limit at different pressures. Minimum detection limits at a) 1 kPa, b) 10 kPa, and c) 100 kPa.


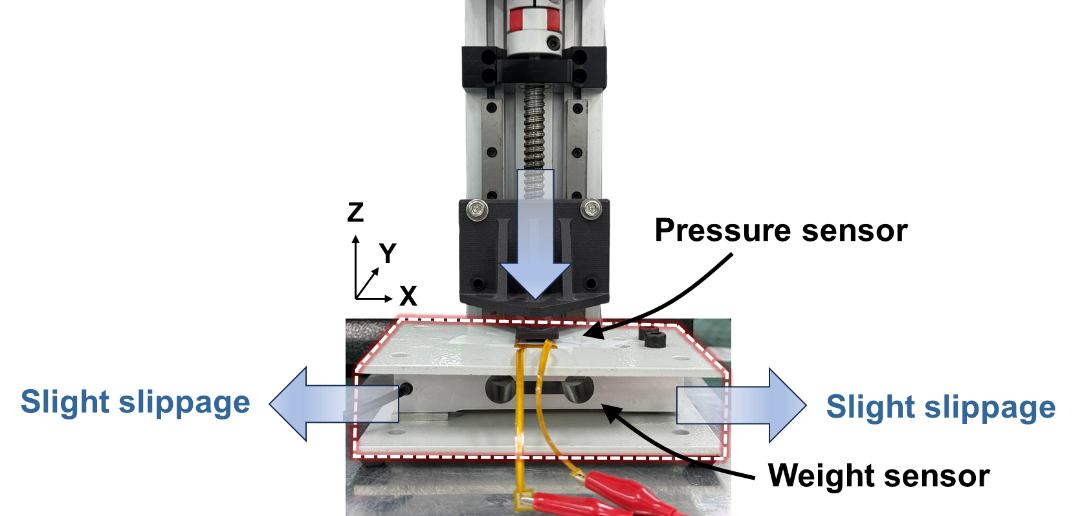


Figure S14. Sensor calibration test system.


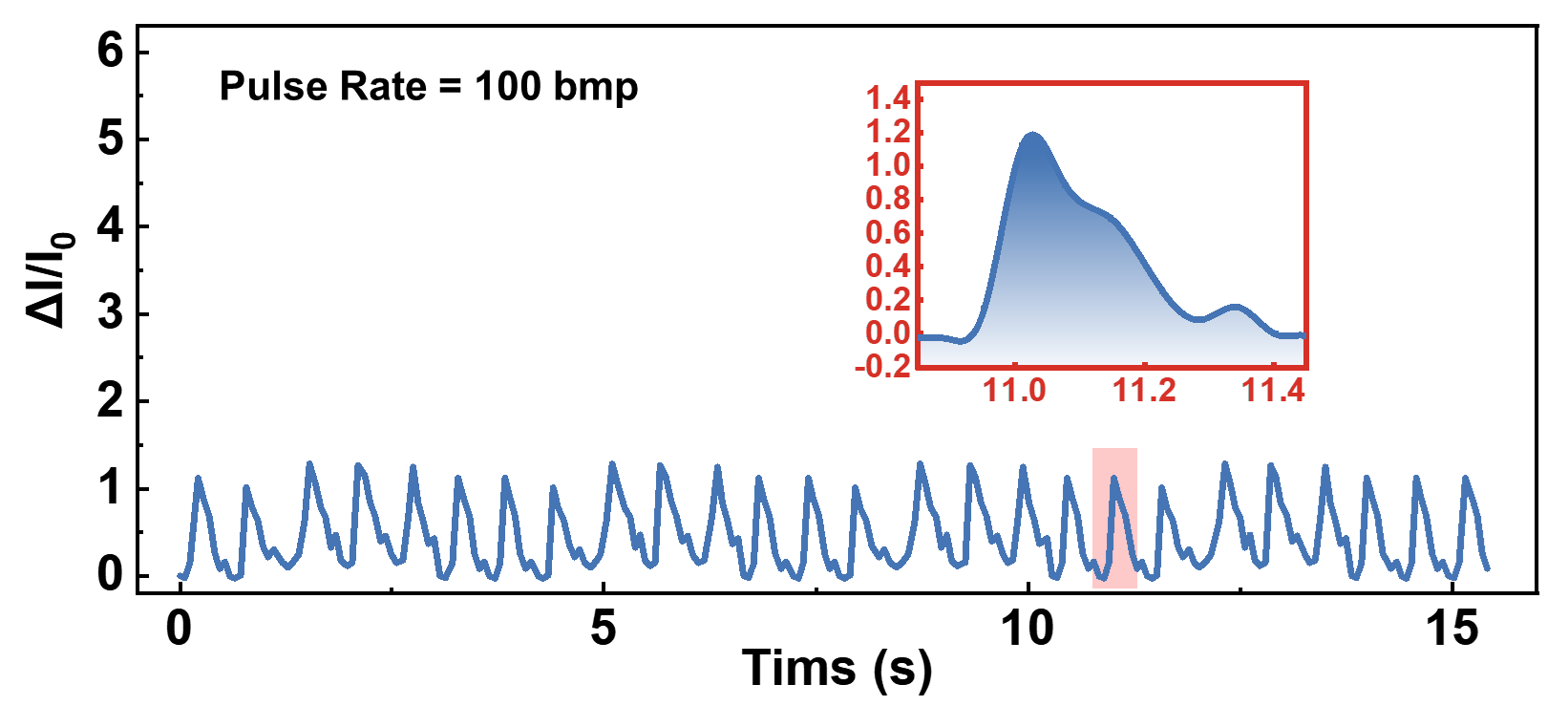


Figure S15. Pulse signal monitoring at wrist.


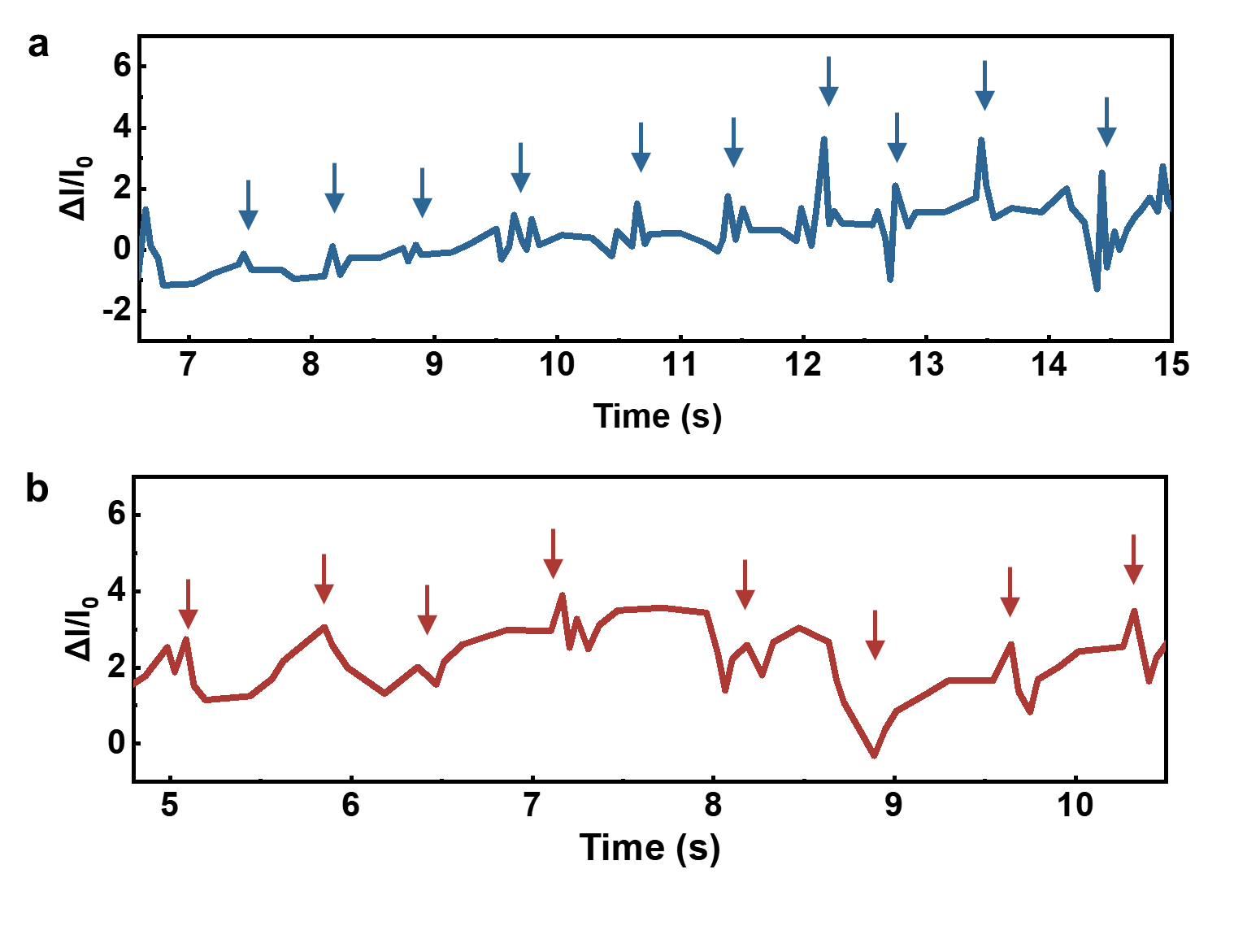


Figure S16. The weak vibration generated by the neck pulse detected by the 3D-OTGM sensor. a) “PolyU” sound monitoring signal. b) “Sensor” sound monitoring signal.


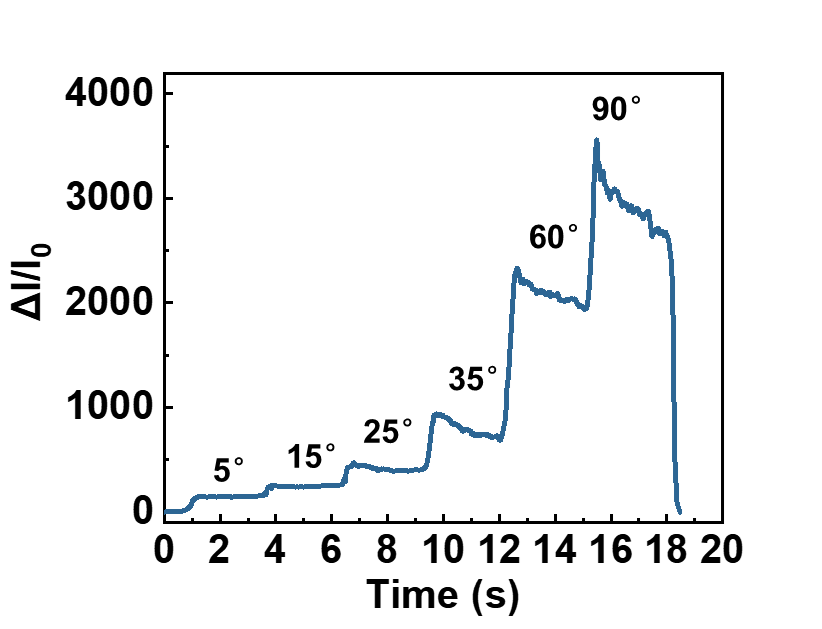


Figure S17. Monitoring of finger joint bending angle.


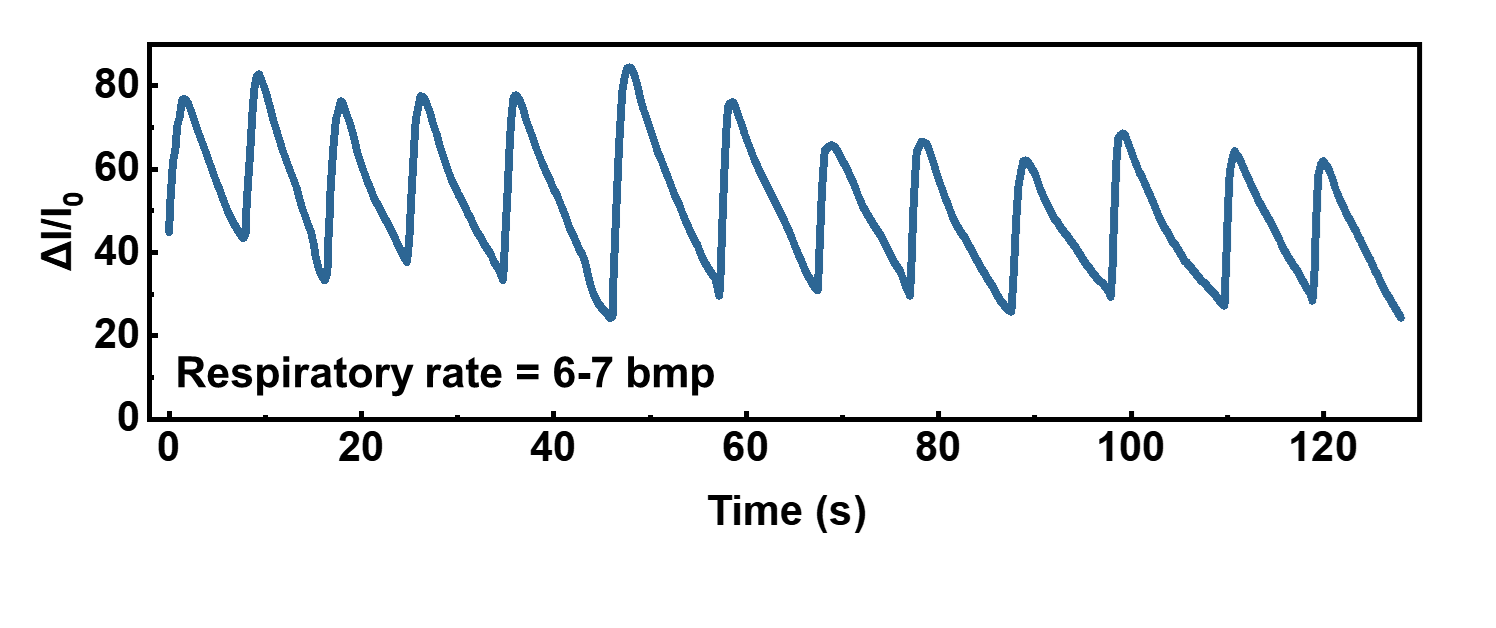


Figure S18. Respiratory rate monitored by 3D-OTGM sensor.

Table S1. Laser method fabricating parameters.

| Grade | Power  (W) | Scanning spacing  (mm) | Scanning speed  (mm/s) | Scanning method | Scanning times |
| --- | --- | --- | --- | --- | --- |
| 1# (40 μm) | 4.6 | 0.01 | 2000 | Interlaced scanning | 1 |
| 2# (240 μm) | 4.6 | 0.007 | 800 | Interlaced scanning | 2 |
| 3# (640 μm) | 4.6 | 0.007 | 800 | Interlaced scanning | 5 |
